# The Application of Nanoparticles in Diagnosis and Treatment of Kidney Diseases

**DOI:** 10.3390/ijms23010131

**Published:** 2021-12-23

**Authors:** Patrycja Paluszkiewicz, Adrian Martuszewski, Natalia Zaręba, Kamila Wala, Mirosław Banasik, Marta Kepinska

**Affiliations:** 1 Department of Emergency Medical Service, Wroclaw Medical University, Bartla 5, 50-367 Wroclaw, Poland; patrycja.paluszkiewicz@student.umw.edu.pl; 2 Department of Population Health, Division of Environmental Health and Occupational Medicine, Wroclaw Medical University, Mikulicza-Radeckiego 7, 50-368 Wroclaw, Poland; adrian.martuszewski@student.umw.edu.pl; 3 Department of Pharmaceutical Biochemistry, Division of Biomedical and Environmental Analysis, Faculty of Pharmacy, Wroclaw Medical University, Borowska 211a, 50-556 Wrocław, Poland; natalia.zareba@umw.edu.pl; 4 Faculty of Medicine, Wroclaw Medical University, Pasteura 1, 50-367 Wroclaw, Poland; kamila.wala.01@gmail.com; 5 Department of Nephrology and Transplantation Medicine, Wroclaw Medical University, Borowska 213, 50-556 Wroclaw, Poland

**Keywords:** nanotechnology, renal, KTx, transplantation, nanomaterial, SPIO, nanostructures, nanomedicine

## Abstract

Nanomedicine is currently showing great promise for new methods of diagnosing and treating many diseases, particularly in kidney disease and transplantation. The unique properties of nanoparticles arise from the diversity of size effects, used to design targeted nanoparticles for specific cells or tissues, taking renal clearance and tubular secretion mechanisms into account. The design of surface particles on nanoparticles offers a wide range of possibilities, among which antibodies play an important role. Nanoparticles find applications in encapsulated drug delivery systems containing immunosuppressants and other drugs, in imaging, gene therapies and many other branches of medicine. They have the potential to revolutionize kidney transplantation by reducing and preventing ischemia–reperfusion injury, more efficiently delivering drugs to the graft site while avoiding systemic effects, accurately localizing and visualising the diseased site and enabling continuous monitoring of graft function. So far, there are known nanoparticles with no toxic effects on human tissue, although further studies are still needed to confirm their safety.

## 1. Introduction

Nanotechnology is an interdisciplinary concept that connects physics, chemistry, biology, electronics, biomedical and materials science, thanks to which its application is very wide [1,2]. Formally, it is based on the design, characterisation, production and applications of materials at the nanoscale (1–100 nm). Still, in practice, the nanoscale is not decisive, but rather the variety of size effects and the resulting material properties [3]. Interest in nanotechnology grew when it was proved that the scale of the materials’ size and shape could determine their physiochemical properties; for instance, a high surface area to the volume ratio, increased reactivity or stability in a chemical process or enhanced mechanical strength of a material. This unique characteristic, which is not found in macromolecules, allows for its completely novel applications [4,5,6]. Nanoparticles (NPs) have found applications in drug design, manufacturing, and diagnostics. When properly designed, they can improve detection selectivity and sensitivity, as imaging agents, delivery systems for encapsulated drugs, proteins and nucleic acids. NPs show high loading capacity, stability, high drug bioavailability and biocompatibility. To target specific cells or organs, ligands such as antibodies, proteins and nucleic acids can be placed on the surface of NPs. The mentioned properties are promising for imaging, creating biomarkers for detecting of various diseases and cells, gene therapies, drug delivery and tissue regeneration [7]. In addition, nanomaterials find application in dentistry, including implantology, periodontology as well as aesthetic and prevention dentistry. Nanomaterials (NMs) such as nano-hydroxyapatite can be used as a coating material for titanium implants, a remineralising therapeutic agent for dentinal hypersensitivity, or a bone-regenerating material [8]. NPs appear to be of particular importance in the kidney because of their potential to deliver drugs directly to the site of diseased tissue, the need for long-term drug supplementation and the low side effects compared to standard therapies. The NP size influences its penetration into the kidney via glomerular filtration or tubular secretion mechanisms, which may represent a breakthrough in the targeted treatment and diagnosis of renal diseases [9]. Furthermore, NPs may become crucial for non-invasive and early detection of the onset of graft rejection, monitoring graft function or even evaluating the transplanted organ [10,11]. The possibilities of using NPs in medicine appear endless. Nanomedicine is gaining more and interest, and numerous scientific reports create information chaos. In our work, we set out to summarise and organise the knowledge on the application of NPs in kidney disease and kidney transplantation. We aim to identify future directions of nanomedicine and the limitations that researchers may face.

## 2. Materials and Methods

A literature review was conducted. Editorial comments, letters to the editor or indexed abstracts at international congresses were not considered. A comprehensive review was performed by systematically analysing the literature published up to September 2021. The PubMed (https://www.ncbi.nlm.nih.gov/pubmed/, accessed on 30 September 2021) and Google Scholar (https://scholar.google.com/, accessed on 30 September 2021) databases were searched for variations of keywords: ‘kidney’, ‘nanoparticles’, ‘drug delivery’, ‘nanotechnology’, ‘transplantation’, ‘nanomedicine’, ‘renal’, ‘theranostic’, ‘encapsulated’, ‘nanoscale’, ‘nanomaterials’, ‘graft function’, ‘disease’ and ‘application’. We also used PubMed Advanced Search Builder to search for the following phrases: (nanomedicine) AND (imaging OR therapy) AND (application); (((nanoparticles OR nanomaterial*) AND (nanoscale)) AND (properties)) AND (nanomedicine); (nanoparticl*) AND (kidney transplantation) AND (kidney diseas*) and others.

**Figure 1:**
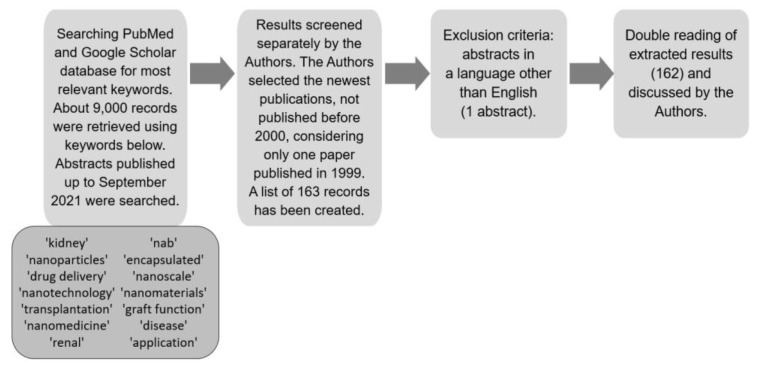
Strategy of data collection presented as a flow diagram.

The literature search was performed by two authors. We found about 9000 records that were screened by the authors. We included 162 papers, which were mainly the newest ones but which specifically concerned kidneys and kidney transplantation. Abstracts in a language other than English were excluded. Publication review included deceased and live donor types, cellular, animal and human studies. Prospective and retrospective clinical studies including center studies, meta-analyses and review articles were included. Formal institutional review board (IRB) approval for this study was not required. The search strategy is presented in Figure 1.

## 3. Properties of Nanoparticles

**Figure 2:**
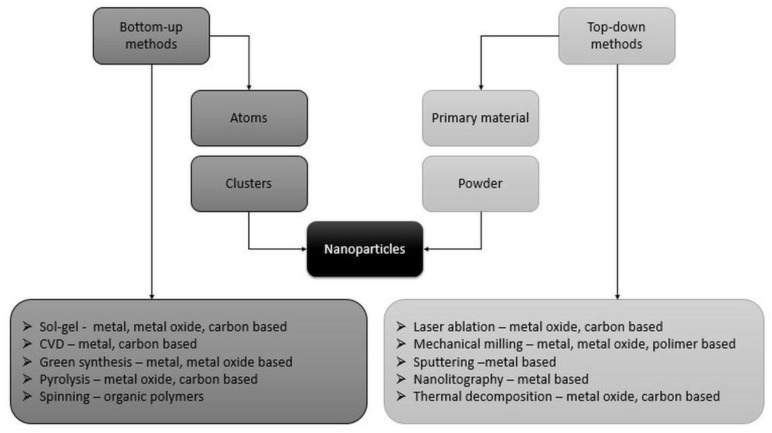
Diagram of nanoparticles synthesis processes and methods and the categories of nanoparticles synthesised from a given method (modified based on Saleh et al. [6]).

NMs are classified as a vast group of materials that have at least one dimension in the nanoscale [6]. Due to the variety of shapes and sizes, NMs can exist in spherical, ellipsoidal, tubular or irregular shapes [12], and can be zero-, one-, two- or three-dimensional [13]. The structure of nanoparticles (NPs), which are NMs in spherical form, includes two or three layers: a core material that defines its key features, a shell material and a surface for intentional functionalisation [14]. The NP synthesis is achieved through many methods, and combinations thereof, that can be generally divided into two groups: bottom-up and top-down approaches (Figure 2) [6].

Size is one of the most important factors that affects NM properties. Gold is a good example of this as it becomes highly active at the nanoscale [15]. It is related to a very high surface area to volume ratio called the surface effect. The percentage of atoms available at the surface of a particle for reaction catalysis will increase with a decreasing radius of the particles [16]. The higher the number of atoms on the surface, the higher the average binding energy per one atom. In turn, the higher energy one atom has, the more it will try to reduce that energy by interacting with the environment [12]. It has been proven that gold NPs with a diameter of 1 micron will have 0.2% of their atoms at the surface, 50 nanometers (nm) in diameter has only 3.4%, while NPs 5 nm in diameter increases this value up to 31% [16]. Thanks to this, the material, which is considered a weak catalyst in the form of bulk material, may have increased catalytic efficiency in the NP form.

Size also determines the optical properties of some NPs. NPs made of noble metals show a strong extinction band in the UV-visible range that does not occur in the bulk metal spectrum [17]. Other significant elements are their shape and structure. By influencing these properties, it is possible to design their features intentionally. The surface charge is connected to the shape and size of the NPs. It determines stability, aggregation, functional group affinity and colloidal behaviour. The context of the last one is critical due to the exposure or influence of NPs on the organism [18].

In order to fully use the enormous potential of NMs, their functionalisation and modification are necessary. The above allows controlling their dispersion, colloidal stability, biocompatibility or interactions with the surrounding environment. In the biomedical field this is especially important in the context of cellular uptake, targeted drug delivery, biosensing or bioimaging [19]. Often, all these functions can be fulfilled by multifunctional NPs, which may include multiple components into a single system by selecting the appropriate core material or materials, and in case of hybrid particles, properties in conjunction with the functionalised surface [20,21,22]. Surface functionalisation involves adding a chemical functional group that enables the self-organisation and compatibility of NPs. This allows creating features such as the surface charge and energy, topology and bioreactivity for the desired application [23]. Functionalisation of the surface is most often carried out by an in situ synthesis, followed by further modification, using inorganic materials, polymers, biomolecules and surfactants as ligands. The synthesis methods enabling surface functionalisation include chemisorption and physisorption, electrostatic, covalent and non-covalent interactions and intrinsic surface engineering [19].

Therefore, these methods can be employed to achieve biocompatibility, dispersibility, reactivity, binding capacity and catalytic activity improvement [24,25].

## 4. Medical Applications of Nanomaterials

**Figure 3:**
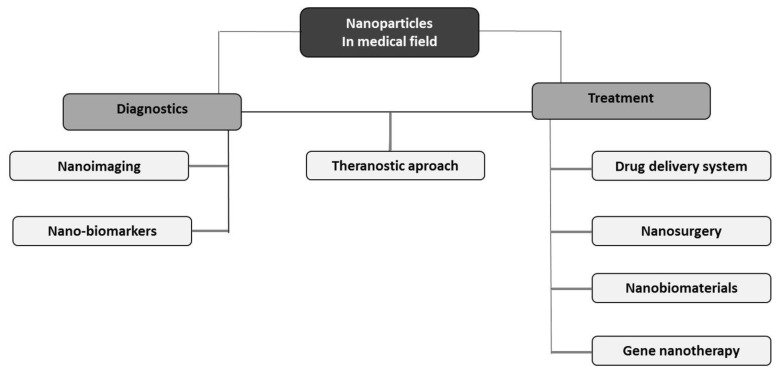
Diagram of the use of nanotechnology in various fields of medicine.

The development of nanotechnology has given hope for new possibilities in medicine. Currently, NMs have various medical applications and are used both to diagnose and treat diseases. With technological advancement and new clinical trials, the indications for using NPs in this field are constantly expanding [26]. Small size, shape, unique mechanical, electrical and optical properties, as well as a large variety of NP formulations allowed for their extensive use in many fields of medical science (Figure 3). 

### 4.1. Nanoimaging and Detection of Various Disorders

Non-invasive imaging techniques has long been one of the most important component of patient diagnostics. Continuous efforts are made to improve image quality while minimizing side effects. Depending on the physical properties of NMs, they can be used as a contrast agent for various imaging methods. Inorganic NMs containing metals such as gold, silver or platinum, and magnetic NPs, can be used as diagnostic tools for tissue imaging [27]. Several nanoparticle preparations and complexes are currently approved for clinical use as magnetic resonance imaging (MRI) contrast agents. As T1 contrast agents, NPs using transition and lanthanide metals (the most important was the ion gadolinium) and iron oxide nanoparticles were used. They increased the longitudinal relaxation times. The newer T1 contrast agents are created by gadolinium complexes immobilized in various nanostructured materials. Contrast agents with iron oxide nanoparticles larger than 50 nm have the ability to shorten the T2 relaxation times, while those smaller than 50 nm image lymph nodes. The use of iron oxide nanoparticles can enhance T1 relaxation (spin-lattice relaxation) times and image the specific site for their accumulation. Nanoparticles used as contrast agents increase the contrast and imaging accuracy [28,29]. Importantly, compared to conventional contrast agents, NPs can be functionalised with molecules on the surface, e.g., with antibodies, and provide better imaging of target tissues. In a clinical trial involving patients with breast cancer, it was shown that superparamagnetic iron oxide (SPIO) NPs could be successfully used for detecting sentinel lymph nodes through magnetic resonance visualisation, which is a good alternative to conventional nuclear medicine techniques [30,31]. Gold and bismuth sulphide NPs can be a favourable replacement for current iodine-based contrast agents in tissue imaging with computed tomography (CT) [32]. In turn, the unique optical properties of quantum dots can be applied in fluorescence-based optical imaging [33]. Other fluorescent agent are silica NPs, suitable for confocal imaging [34]. Molecular imaging with ultrasound uses nanobubbles with a gas core. Due to the size of nanobubbles (100–1000 nm), they can overcome the endothelial barrier, extravasate, and target pathological tissues, e.g., tumour areas [35]. In preclinical studies, functionalised PSMA- or CA-125 antibody-conjugated nanobubbles showed promising results in diagnosing prostate and ovarian cancer [36,37].

Superparamagnetic iron oxide (SPIO) nanoparticles may be useful for in vivo studies targeting mononuclear cells of the phagocytic system. They are phagocytosed by the Kupffer cells in the liver. They can be used to diagnose intrapancreatic accessory spleen and exclude haemangiomas or cysts [38,39]. In addition, SPIO-enhanced MRI can help detect the localisation of macrophages in the diagnosis of abdominal aortic aneurysms. The authors found that macrophages endocytosed SPIO contrast agents [40].

Ultra-small particles of iron oxide (USPIO) are macromolecular agents whose structure is based on iron and has a longer blood half-life [41]. USPIO NPs can also be a marker of the inflammatory process through the detection of macrophages. Therefore, they have clinical implications for cardiology in diagnosing atherosclerosis-associated inflammation before visible narrowing of the arteries [42,43]. Additionally, USPIO accumulates in myocardial infiltrating macrophages, which is the basis for USPIO-MRI in diagnosing conditions such as myocarditis, chronic ischaemic cardiomyopathy and infarction [44]. Pathogen detection using nanotechnology is considered rapid, accessible and very effective due to its high sensitivity. Nanomaterials with proven efficacy in detecting infections (viral and bacterial) include graphene-based materials, quantum dots (QDs) and gold or magnetic NPs [45]. For instance, QDs can be used to distinguish the *respiratory syncytial virus* (RSV), *Hepatitis B Virus* (HBV), *Hepatitis C Virus* (HCV), *Human Immunodeficiency Virus* (HIV), or bacteria such as *Escherichia coli* or *Mycobacterium* [46]. In turn, gold NPs combined with antibodies or DNA have wide use in diagnosing viral infections caused by *Flaviviridae*, *Coronaviridae*, *Herpesvirdae*, *Orthomyxoviridae* and others [47]. Recently, due to the coronavirus disease 2019 (COVID-19) pandemic, a lot of research has been carried out into using gold NPs functionalised with aptamer, antibodies or DNA/RNA oligonucleotides to detect severe acute respiratory syndrome coronavirus 2 (SARS-CoV-2) infection [48,49,50]. In addition, gold NPs functionalised with short antigenic epitopes are also useful in serodiagnosis because they detect the presence of SARS-CoV-2 IgG antibodies, which allow assessing the humoral response to infection or the effectiveness of vaccination [51]. 

### 4.2. Drug Delivery System

One of the primary and commonly used functions of NPs is the drug delivery system. Due to their hydrophobicity or instability, some active agents show insufficient bioavailability. NPs are utilised as carriers for some drugs and biological macromolecules (such as peptides, proteins and nucleic acids) improving the pharmacokinetic properties of these molecules [52]. Paclitaxel (Ptx), a highly hydrophobic and water-insoluble taxane, is administered to patients in a solution containing ethanol and polyoxyethylated castor oil, which can cause increased frequency of hypersensitivity reactions and often requires premedication with antihistamines and steroids [53]. Specially designed NPs improve the bioavailability of Ptx. For instance, the albumin-bound, 130 nm particle formulation of Ptx (nanoparticle albumin-bound paclitaxel, nab-Ptx) shows increased solubility in water and allows preparing a drug solution based on physiological saline without using toxic solvents. In addition, it has a larger fraction of the free form of the drug, which improves response to treatment [54]. Clinical trials in patients with breast cancer demonstrated a statistically significant increased rate of complete pathological remission with nab-Ptx compared to paclitaxel alone [55,56]. The use of nab-Ptx was also well-tolerated and improved overall survival in patients with pancreatic cancer, advanced squamous cell carcinoma of the head and neck, or advanced squamous non-small cell lung cancer [57,58,59]. 

Drug encapsulation in liposomal NPs prevents untimely drug action before reaching the targeted site, reducing toxicity [60]. In addition, the application of NPs in oncological therapy limits adverse effects of cytotoxic drugs, particularly nausea, vomiting, neutropenia and hypersensitivity reactions [61]. For example, combining doxorubicin (DOX) with liposomal NPs decreases the cardiotoxicity of the anthracycline with preserved antitumor activity in patients with metastatic breast cancer [62,63].

NPs acting as a carrier for DOX, an anticancer drug in breast cancer, reduce the adverse effects of pro/antioxidant imbalances in cancer and non-cancer cells. The application of fullerene C_60_ as a carrier for DOX reduces drug-induced metallothionein levels and decreases superoxide dismutase activity. Furthermore, therapy combining NPs with DOX is as effective as with DOX alone. Exposure of breast cancer cells to the DOX–C_60_ complex decreased their number [64]. Metal–organic framework NPs with DOX or lipid nanocarriers with Ptx also showed improved safety profiles in treating breast or ovarian cancer [65,66]. Fullerenes may also be a promising nanotransporter for drugs. It was proven that C_60_–DOX complexes increase the cytotoxicity of antineoplastic drugs against breast cancer cells. Interestingly, DOX was shown to be released from C_60_ at a lower pH, potentially providing less toxicity to healthy tissues with a less acidic environment [64,67]. Furthermore, the ability to bind ligands such as antibodies to nanocarriers allows for selective, targeted drug delivery to cells with appropriate receptors on their surface. For example, attaching anti-HER2 antibodies to NPs increases the uptake of the molecule by HER2-overexpressing cells, which may enhance the effectiveness of cancer treatment, while reducing systemic adverse effects [68,69]. The nanocarrier system also increases the effectiveness of the treatment of infectious diseases caused by bacteria, fungi and viruses. The active antimicrobial components combined with NPs include ketoconazole, tobramycin, ciprofloxacin or rifampicin. The advantages of this transport system include increased bioavailability and physical stability or prolonged drug release, which maintains the high antimicrobial activity of the chemotherapeutics [70]. Interestingly, it was shown that silver NPs themselves have bactericidal properties (antibacterial activity was proven, e.g., for *Staphylococcus aureus* and *Escherichia coli*) [71]. In vitro studies involving the delivery of amantadine or oseltamivir combined with selenium NPs in the treatment of influenza virus infection showed promising results in overcoming the common problem of drug resistance [72,73]. In addition to drug delivery, ligand-functionalised NPs with immunomodulatory properties may be beneficial in the treatment of tuberculosis or HIV infection, e.g., by enhancing the secretion of reactive oxygen and pro-inflammatory cytokines, stimulating the immune response [74]. Due to the size of NPs and the possibility of crossing the blood–brain barrier, nanocarriers seem to be also a promising solution for the treatment of CNS diseases. The nano-drug delivery system includes delivering to the CNS, e.g., dopamine, nerve growth factor or neuroprotective peptide in neurodegenerative diseases, adenosine after stroke, or anti-cancer agents such as temozolomide [75,76].

Another method of delivering substances to cells is through lipoplexes, defined as complexes of cationic liposomes and DNA. Lipoplexes serve as non-viral gene carriers. Importantly, lipoplexes can be administered in a variety of routes, including intravenous, transdermal, subcutaneous or inhalation. Their effectiveness has been proven, among others in oncology, ophthalmology and in the treatment of cystic fibrosis [77,78]. Lin et al. [79] modified these complexes by combining cationic lipids with an anti-tumour antibody, Herceptin, which binds to HER2-positive breast cancer cells. By loading such carriers with anti-cancer drugs, they created a drug delivery system customized for the treatment of breast cancer in those with a positive HER2 status.

Recently, layer-by-layer (LbL) assembled drug delivery systems have also been designed. This platform enables the targeted delivery and controlled release of anti-cancer drugs. LbL has been shown to be effective in the treatment of, among others, breast, ovarian and pancreatic cancers as well as melanoma. The release of neoplastic drugs from the LbL system can be regulated in various ways—by changing the light intensity, temperature or pH. Target delivery of substances can also be controlled by various molecules that make up the surface of the LbL system, which are designed to bind to proteins on the surface of cancer cells [80].

### 4.3. Theranostic Approach

Currently, many researchers are trying to use the potential of NMs for theranostic purposes, which are therapeutic and diagnostic at the same time [81]. The theranostic approach includes visualising tissues, image-guided drug delivery using MRI, CT, ultrasound or optical imaging, targeted, local drug release, as well as monitoring drug biodistribution and assessing of therapeutic efficacy [82]. Functionalised metal NPs can accumulate in the tumour area, providing imaging of cancer cells and image-guided drug delivery, including controlled, targeted drug release [83]. In preclinical studies, the theranostic strategy concerning gadolinium-based NPs combined with radiation therapy or anticancer agents revealed a promising, synergistic effect in oncological treatment [84,85,86]. Clinical trials in patients with brain metastases demonstrated the effectiveness of gadolinium-based NPs in increasing MRI contrast and a favourable safety profile. However, evaluating the effects of radiation sensitisation is still in progress [87]. In addition, nanotechnology is applied to visualise brain tissue while attempting to cross the blood-brain barrier to deliver drugs in central nervous system (CNS) diseases. Microbubbles (MBs) are gas-filled vesicles ranging from 1 to 100 µm most commonly used as contrast agents for ultrasound imaging, which also perform well as a drug delivery system and facilitate sonoporation by increasing cell membrane permeability. It was noted that MBs loaded with USPIO NPs enable the modification of the permeability of the blood–brain barrier using ultrasound pulses and the monitoring of drug delivery to the CNS with MRI [88]. As previously mentioned, iron oxide-based NPs can be used to diagnose cardiovascular pathologies. Combining this nanomaterial with fibrinolytic drugs enables both the assessment of atherosclerotic vasoconstriction and the dissolution of clots. Other examples of theranostic approaches using nanotechnology in cardiology are imaging and inhibition of angiogenesis, targeted delivery of stem cells to the infarcted myocardium and nano-mediated drug-eluting stents [89]. Inhibition of angiogenesis is possible through the use of NPs with anti-angiogenic drugs. Winter et al. [90] reported decreased MRI enhancement in vasa vasorum (inhibited expression of ανβ3 integrin) and fewer microvessels after administering rabbits with integrin-targeted paramagnetic NPs with fumagillin (30 µg/kg)**.** After a myocardial infarction, gold or SPIO NPs enable imaging of the post-infarction scar in MRI. At the same time the supplied stem cells (e.g., mesenchymal stem cells, cardiac progenitor cells or cardiac stem cells) provide regeneration and repair of damaged myocardium [91]. Additionally, research on NP drug-eluting stents (NP-DES) in invasive cardiology gives promising results. Biodegradable polymer NPs incorporated with drugs showed better biocompatibility and facilitated prolonged drug release. Moreover, in contrast to sirolimus-eluting stents, no delayed endothelial healing effect was observed in the NP-DES group with retained efficacy [92,93]. 

### 4.4. Nanosurgery and Nanobiomaterials

Nanodevices, including femtosecond laser systems, nano-knives, nanotweezers and nanoneedles, enable procedures at the level of single cells, minimising damage to adjacent tissues [94]. Nanoneedles have the potential to provide precise and safe local delivery of active substances via the transdermal route [95]. Zhu et al. [96] demonstrated that diamond nanoneedle arrays enable the drug delivery directly into the cell’s cytoplasm, increasing the effectiveness of anticancer treatment. Nanotweezers are devices for manipulating nanostructures. In medicine, silicon nanotweezers found application, for example, in manipulating DNA molecules, which facilitated their characterisation [97]. In neurosurgery, the nano-knives, creating a 20 nm incision, became a novel tool for peripheral nerve surgery, allowing for the isolation and cutting of a single axon [98]. The nano-knife appears to be a revolutionary tool in oncology for minimally invasive ablation of solid tumours by irreversible electroporation [99]. 

NMs contributed to the development of surgery, especially neurosurgery and orthopaedics. Titan-based NMs are used in the production of implants due to their excellent biocompatibility, which can reduce postoperative pain and contribute to faster wound healing [100]. NMs found applications also in dentistry. Silver, chitosan, copper oxide or zinc oxide NPs with antibacterial properties are an excellent substrate for composite adhesives. In turn, titanium oxide nanotubes form the surface of the implants [101]. 

Nanotechnology also allows the fabrication of bio-absorbable haemostatic nanomaterials and anti-infectious nanosilver products. Haemostatic materials include chitosan nanocomposite, halloysite nanotube, the nanofibrous membrane of carbonised hair and synthetic mesoporous silica-based xerogels [102]. In turn, nanosilver can be utilised to produce dressings, surgical sutures and implant coatings. In addition, it has an excellent antibacterial effect and, thus, a wide range of clinical applications; however, the toxic effect of nanosilver was noticed [103]. 

### 4.5. Gene Nanotherapy

Another field of medicine, rapidly developing with nanotechnology, is gene therapy, where research focuses on altering gene expression. One of the mechanisms used to change gene expression is the delivery of small interfering RNA (siRNA) to cells by nanocarriers such as lipid NPs [104]. In this case, gene silencing occurs by degrading the target mRNA by siRNA, thereby inhibiting translation [105]. For instance, ALN-VSP (Alnylam, a stable nucleic acid–lipid-based nanoparticle targeting vascular endothelial growth factor), i.e., lipid NPs combined with siRNAs targeting vascular endothelial growth factor (VEGF) and kinesin spindle protein (KSP), downregulate the expression of VEGF and KSP in patients with advanced solid tumours, due to the mechanism of RNA interference. Targeted molecular therapy that changes the expression of certain genes can significantly benefit the treatment of cancerous diseases such as brain tumours and neurodegenerative disorders [106,107,108]. Another benefit from molecular targeted therapy is silencing the gene encoding P-glycoprotein, which is a protein responsible for removing many foreign substances from cells, thus acting as a drug exporter, may overcome the problem of multi-drug resistance [109]. The development of a nanosystem for the simultaneous delivery of siRNA (reducing the expression of P-glycoprotein genes) and an anti-cancer agent using mesoporous silica NPs allows for improving the cytotoxicity of chemotherapeutic agents towards cancer cell lines [110,111]. 

## 5. Nanoparticles in Diagnosis and Treatment of Kidney Diseases

The kidney is a particularly interesting site for the use of nanoparticles in the treatment of kidney diseases due to the sensitivity of this organ to the toxicity of previously used drugs, as well as the mechanism of excretion of various metabolic products and contact with molecules in the blood. Drug toxicity is problematic in kidney transplantation. Kidney structure and mechanisms make kidneys a great target for the use of nanoparticles in reducing drug toxicity in kidney transplantation. The kidney has the innate ability to remove particles smaller than 10 nm rapidly. Such properties are due to the anatomical structure of the glomeruli having a basement membrane, podocytes, a fenestrated endothelium separating the mesangium from the extracellular matrix and the mechanism of glomerular filtration. Exploiting these and other properties of NPs provides new opportunities for the detection, treatment and monitoring of kidney disease. Interest in NPs in the kidney continues to grow and results in more and more novel ideas for their application [112].

### 5.1. Nanoparticles in Kidney Diseases

By September 2021, there were 10 clinical trials registered in the ClinicalTrials.gov database for the use of NPs in kidney diseases, which are included in Table 1. However, there is a need for more trials.

To date, various non-clinical studies have been conducted describing the use of nanoparticles in the diagnosis and treatment of kidney diseases, and in kidney transplantation. We have included the most important of these in Table 2.

#### 5.1.1. Monitoring of Kidney Function and Structure

NPs can be used in CT imaging as contrast to assess renal fibrosis. A study using gold NPs with anti-collagen-I antibody (Co-I-AuNPs) in mice demonstrated that kidneys with renal artery stenosis showed increased retention of Co-I-AuNPs compared with kidneys without renal artery stenosis. Fibrosis is central to ischaemic renal damage caused by renal artery stenosis. Therefore, assessment of renal fibrosis is important for monitoring disease progression, and invasive biopsy is currently the only reference method. Furthermore, no significant changes in haematology, electrolyte, liver and kidney function were observed in mice 24 h after injection of gold NPs, indicating short-term safety [113].

There is growing interest in the renal-clearable luminescent metal NPs in kidney diseases, cancer, and antimicrobial activity. The main organ responsible for the excretion of these NPs is the kidney. The kidney excretion pathway allows for non-invasive monitoring of kidney function using kidney clearance kinetics (KCK) assessment. Non-invasive fluorescence imaging has become difficult due to the high accumulation of conventional fluorophores in the skin. This problem can be solved by using renal-clearable metal NPs with NIR excitation and emission. The glutathione-coated luminescent gold NPs (GS-AuNPs) significantly improved imaging-time window and kidney contrast compared to conventional dyes. Deviations of non-invasive and invasive KCK were minimised. The use of high-contrast and non-invasive fluorescence imaging with GS-AuNPs is promising for preclinical assessment of renal function and may provide a functional marker [116]. 

Ordikhani et al. [117] designed NPs that selectively interact with proximal tubule epithelial cells in the kidney and renal clear cell carcinoma cells, which commonly arise from proximal tubule epithelial cells. Nanocarriers—PEGylated polylactic-coglycolic acid—have lambda light chains on the surface that interact with the membrane protein megalin. The NPs showed no renal toxicity in the mice tested. Megalin expression was demonstrated not only in the primary tumour but also in lymph node metastases, which meant that the NPs accumulated there as well. NPs can provide a contrast agent, increasing the sensitivity of detecting subclinical malignancies and metastases. Light chain-conjugated NPs could help develop drugs targeting proximal tubule epithelial cells, whose damage commonly leads to acute kidney injury due to ischaemia-reperfusion injury, sepsis and drug toxicity.

Ultra-small gold nanoclusters (AuNCs) have exceptional tumour accumulation capacity and efficient renal clearance properties, but the catalytic activity of AuNCs has not been sufficiently studied. Loynachan et al. [128] designed multifunctional protease nanosensors using the peroxidase-mimicking activity of AuNCs, which react at pathologically altered sites. Direct colorimetric reading of urine is obtained in less than an hour and is disease dependent. There was a 13-fold colourimetric increase in the urine of colon cancer mice assessing the catalytic activity of AuNCs compared to healthy mice. Within four weeks, the NPs were eliminated by the liver and excreted by the kidney without signs of toxicity.

#### 5.1.2. Potential Therapeutic Application

Midgley et al. [129] investigated the utility of NPs in delivering anti-fibrotic therapies to damaged cells and tissues. They conducted the study on mice with a narrowed ureter. The mice were administered plasmid DNA expressing bone morphogenetic protein 7 or hepatocyte growth factor by encapsulation within chitosan NPs coated with hyaluronan. The effect of the NPs included improved renal function and halted progression of chronic kidney disease by eliminating the accumulation of collagen fibres, reversing fibrosis and regenerating renal tubules. Gene delivery NP therapy has better bioavailability and activity than cytokine therapy. Further research is needed on the targeted delivery of anti-fibrotic and regenerative genes to renal cells. 

Filtration in the glomerulus occurs due to electrostatic repulsion, which provides permeability for anionic molecules. The properties of the glomerular barrier in renal disease can be used to deliver NPs to specific cells. To reach these cells, NPs have to escape from the general circulation and overcome the filtering barrier in the glomerulus [130]. NPs with antibodies in kidney disease represent an important direction for medical and pharmaceutical development. It is possible to produce antibody-conjugated NPs that deliver therapeutic agents or are used in imaging and are highly specific for selected cells. For example, SPIO NPs conjugated to an anti-CR2 monoclonal antibody bind to complement protein C3 in the relevant inflamed sections of the kidney, and this process can be visualised using MRI. Dexamethasone can be delivered to glomerular endothelial cells (ECs) in liposomes targeting E-selectin, which reduces inflammation in glomerulonephritis, counteracts disease progression and reduces kidney damage. Iron oxide NPs conjugated to the anti-vascular cell adhesion molecule-1 (anti-VCAM-1) antibody bind rapidly to the endothelium and can deliver drugs and imaging agents to this site. Vascular cell adhesion molecule-1 (VCAM-1) expression increases in the kidney ischemia–reperfusion injury [114]. Kidney ischemia–reperfusion injury is an important pathological process in transplantation, among others. Adhesion molecules on the surface of the vascular endothelium persist after cessation of ischaemia, so endothelial cell-specific iron oxide NPs seen on magnetic resonance imaging may carry information about the extent of the injury and be used to implement appropriate treatment [131]. 

Focal segmental glomerulosclerosis (FSGS) is a process in which loss of renal podocytes and progressive scarring of the glomeruli occur. It is one of the main glomerular causes of chronic kidney disease and its treatment is based on glucocorticosteroid therapy with various side effects. NP drug delivery systems can overcome the previous problems by tailoring the pharmacological profile of the drug load taking into account the NP size, composition, charge and ligands such as peptides, antibodies and aptamers. Previous studies showed that NPs of 5–30 nm size were readily detected in urine without significant accumulation in the kidney; NPs of 60–100 nm in size accumulated in the basement membrane of the glomerulus and NPs of 75 nm in size collected in the mesangium. It is crucial that in glomerular disease, increased permeability of the filtration barrier can alter the kinetics of NP drug delivery, and the glomerular disease itself is usually accompanied by injury and inflammation. Increased expression of receptors on the surface of leaky blood vessels and disease entity-specific enzymes can be exploited to accumulate appropriately engineered NPs and release drugs at the diseased site [132]. 

Williams et al. [115] conducted a study on mice administered mesoscale NPs (MNPs). To improve kidney selectivity, the administration route was changed, and the dose was adjusted. After intravenous administration, MNPs localised with 5–7-fold higher efficacy in the kidney than in other organs, particularly in the renal tubular epithelium, and persisted for up to 7 days. Long-term biodistribution studies were also performed, which excluded the negative consequences of increased accumulation of MNPs in renal tubules on renal function and the body as a whole. Furthermore, renal targeting was shown not to be dependent on enclosed cargo, and MNPs can deliver hydrophobic and hydrophilic cargoes of small and larger molecules (e.g., Cy5-labelled dsDNA) to the kidney. 

Nanoencapsulation is extremely promising because the coating of NPs containing nephrotoxic drugs (e.g., anti-inflammatory drugs, chemotherapeutics) is not harmful. Such NPs can release drugs in a controlled manner and target renal cells. Fluorophores on the surface of NPs can also emit separate and intense fluorescence to kidney cancer cells. Such NPs are anchored to the cancer tumour using an antibody that binds to antigens on the surface of the cancer cells. The drug is a chemotherapeutic agent and its effect is enhanced by the laser beam [133]. 

A rapidly growing branch of nanotechnology is cancer diagnosis and treatment. CuO induces oxidative stress, cell cycle inhibition, antioxidant depletion and Zn-induced cancer cell apoptosis, resulting in kidney cancer cell death. Zn NPs also have a high potential to kill cancer cells. Xue et al. [118] studied the toxicity effect of Zn/CuO on normal human epithelial cells (HK-2) and A-498 kidney cancer cells. Zn-doped CuO NPs are more toxic to kidney cancer, which is due to the CuO NPs killing by Zn ions. The study shows that Zn/CuO induces excellent inhibition of renal cancer cells but does not affect the viability of normal renal epithelial cells. 

### 5.2. Nanoparticles in Kidney Transplants

After organ transplantation, the main clinical problem is rejection, which requires lifelong immunosuppression, accompanied by susceptibility to infections and cancer. One of our works describes the potential use of cell-free DNA in monitoring patients after transplantation [134]. There is also a necessity for proper qualification of donors and prediction of graft function after kidney transplantation (KTx).

#### 5.2.1. Prevention of Transplant Rejection

Currently, the gold standard for assessing kidney function is an invasive biopsy. Non-invasive markers are needed to allow continuous monitoring of transplanted organ function. In addition to metabolites such as creatinine, hippuran, mannitol and alanine [135], as well as donor-specific antibodies [136,137,138], appropriately designed NPs are promising markers. Mac et al. [10] developed NPs for early rejection detection in urine. The study was conducted in mice given NPs conjugated to a peptide substrate specific for the serine protease granzyme B. It is produced during acute rejection (AR) by the recipient’s T cells, so as a non-invasive marker of early rejection, it may offer an alternative to invasive biopsy. After systemic administration, the nanosensors accumulated in allograft tissue and then were cleaved by granzyme B, which released a fluorescent reporter that penetrated the recipient’s urine. Urine analysis determined the onset of rejection with high sensitivity and specificity.

Nanotherapeutics hold promise for achieving effective transplant tolerance by replacing cell-based therapies due to advantages such as reproducibility, ease of storage and lower cost. The phenomenon of biomimicry is becoming increasingly important in the development of new tolerance generation technologies. Polymer-based NPs are an attractive carrier platform for modulating the immune response in antigen-specific diseases. In transplantation tolerance, they deliver immunosuppressive drugs in a targeted manner, avoiding systemic toxicity. Lipid-based NPs are more promising than polymer-based NPs due to their higher biomimicry. They are attractive carriers of plasmid DNA or siRNA directly into a cell that needs to reprogram genetic material to elicit a long-term response. Liposomes can also deliver immunosuppressive drugs, such as cyclosporine or tacrolimus, to allografts. Donor exosomes secreted by transplant cells were shown to be natural NPs and to modulate the immune response, initiating a direct alloreactive T-cell response shortly after transplantation. Such extracellular vesicles have the potential to provide signals for the effective activation of specific immune responses. Another method using NPs for tolerance includes hybrid models using synthetic molecules coated with cell-derived components. They are designed to deliver donor antigens inducing graft tolerance [119]. 

Delayed graft function (DGF) contributes to short-term and long-term graft failure. Thrombin-targeted perfluorocarbon NPs (PFC-NP) protect against ischaemic kidney damage after transient arterial occlusion. Vemuri et al. [120] set out to investigate whether perfusion of allografts with PFC-NP protects the transplanted kidney after a period of ischaemia. The study was conducted on rats that received a kidney transplant after 6 h of refrigerated storage. Compared with the control group, serum creatinine levels were improved, histological kidney damage was smaller, and Kaplan–Meier survival curves indicated increased longevity. In addition, NP-based antithrombin showed a protective effect on blood vessels and enhanced allograft function. 

The first site of contact between the donor tissue and recipient immune cells in the circulation is the vascular endothelium. Rejection of a vascularised allograft can have a cellular and humoral mechanism, both of which are directed against alloantigens of the ECs of the allograft blood vessels. Contact between recipient immune cells and graft alloantigens first occurs during reperfusion, from which point the immune response is driven. Brasile et al. [124] attempted to develop an effective organ-specific therapy to protect allograft vessels from immune-mediated injury. In their previous study, they designed an immunocloaking membrane for renal vessels covering luminal surfaces. The membrane contained laminins, glycoaminoglycans, collagens and fibronectin applied to the allograft during normothermic perfusion, and provided 90% coverage of the luminal surfaces of the renal vessels. Leukocyte transmission occurred only at the site of direct contact with the blood vessels. The ImmunoCloak membrane interrupts contact between leukocytes and blood vessels and inhibits endothelial interactions, leading to the passage of immune cells into the graft. Such protection lasts approximately 21 days and is, therefore, a short-term therapy. Thus, Brasile et al. [124] demonstrated that ImmunoCloak interrupts antigen presentation, disrupts diapedesis and prevents early T-cell activation, with leukocyte migration through endothelial cell layers reduced by 93%. The synthesis of pro-inflammatory cytokines was significantly inhibited, and the T cell-mediated response was blocked. Membrane therapy may obviate the need for nephrotoxic immunosuppressive drugs in the early post-transplant period, alleviate the degree of delayed graft function and allow the use of more ischaemic renal allografts.

The use of immunosuppressive agents (ISAs) has reduced the overall rate of acute graft rejection, but carries complications in the form of infections, cardiovascular disease, diabetes and malignancies. There is also a need to improve long-term transplant outcomes. There are inflammatory reactions within the allograft that significantly increase alloimmunisation. Significant expression of inflammatory cytokines and adhesion molecules occurs at the time of brain death and reaches its peak after the anastomosis of the transplanted organ and coincides with ischemia–reperfusion injury. Alloreactive T cell responses exacerbate the chronic alloimmune injury. Therefore, control of immune-activating factors to improve transplant outcomes is essential. Uehara et al. [121] developed an NP carrier of mycophenolate mofetil (MMF), a drug that inhibits purine metabolism in lymphocytes, prevents their proliferation, and has an excellent safety profile. Direct delivery of ISAs to the organ at the time of transplantation reduces intra-graft inflammation and alloimmune activation at a critical time. The targeted delivery and sustained release MMF system developed by Uehara et al. [121] is applied for pre-transplant treatment. 

The site of occurrence and recognition of alloantigens are the draining lymph nodes, which generate activated and intensely proliferating T lymphocytes responsible for acute graft rejection. Targeted delivery of immunosuppressive drugs to the lymph nodes can alleviate graft rejection. Highly effective is tacrolimus (TAC), which inhibits interleukin-2 production, suppresses T-cell activation and even induces T-cell apoptosis. In addition, NPs can improve drug solubility in water, increase circulation time, decrease systemic toxicity and increase accumulation in the diseased site. Human serum albumin (HSA) used as a nanocarrier seems promising. Zhang et al. [122] developed a delivery system formulation of HSA-based formulation of TAC using heart transplants in mice. The safety profile of the HSA-based formulation of TAC was found to be higher, with a lower increase in creatinine concentration compared to the free TAC group and a reduction in drug accumulation in the kidney. 

Porous silicon NPs (pSiNPs) provide an alternative delivery method for immunosuppressive drugs and target dendritic cells. Stead et al. [123] conducted a study in mice using rapamycin and ovalbumin peptide-loaded pSiNPs to assess the ability to generate murine CD4+CD25+FoxP3+ regulatory T-cells. The pSiNP uptake by splenic and peripheral blood dendritic cells was confirmed by flow cytometry. In ovalbumin-sensitised mice, the number of regulatory T-cells was five-fold higher after administering rapamycin and ovalbumin peptide-loaded pSiNP with the monoclonal antibody CD11c compared to the control group. Immobilised antibodies are of great importance in enhancing cellular uptake and generating splenic regulatory T cells. Furthermore, pSiNPs containing monoclonal antibodies targeting dendritic cells are well tolerated and show enhanced kidney-targeting ability of DC-targeting pSiNPs. The ability of pSiNPs to target drug delivery, chemotherapeutics and genomes can be used to treat various kidney diseases, such as Fanconi syndrome, kidney cancer and cystinuria. Given the findings that pSiNP can modify dendritic cells, this therapy may also be a targeted treatment in transplantation and other immune-mediated diseases. 

NMP increases the number of available organs for transplantation by resuscitating organs that could be rejected. The delivery by machine perfusion of drugs that act directly on vascular ECs is a major goal for treating and reducing perioperative damage to ECs. Anti-CD31 antibodies on the surface of NPs were shown to increase targeting to ECs of the transplanted kidney. Studies also demonstrated the therapeutic potential for targeted NP delivery during NMP [139].

#### 5.2.2. Inhibition of Premature Transplant Insufficiency Because of Nonimmunological Factors

The study performed on rats by Xie et al. [125] was designed to assess the effect of Berberine NPs (BBR-NP) on kidney tubular epithelial cell damage due to ischaemia and reperfusion—one of the most common causes of acute kidney injury (AKI). Both Berberine and BBR-NP protected rat kidneys from functional deterioration and morphological damage, although a better effect was observed when BBR-NPs were used. The mechanism of action of BBR-NP was elucidated based on the reduced expression of oxidative and mitochondrial stress pathway proteins. Oxidative stress is reversed, and renal cell apoptosis is inhibited. The protective effect of BBR-NPs offers hope for new therapies for the so far poor prognostic ischaemia–reperfusion injury. 

Cerium oxide NPs (CNPs) have been used to treat acute kidney injury. The systemic inflammatory response caused by oxidative stress is mainly responsible for AKI due to intra-abdominal infection. CNPs decrease caspase-3 activity, remove reactive oxygen species, reduces tubular damage and concentrations of kidney damage biomarkers and improves renal function [126].

Important in achieving improvements in early and long-term graft function and survival is the treatment of kidneys transplanted during the preservation interval. Functional preservation platforms for treating these kidneys can be hypothermic machine perfusion (HMP) and normothermic machine perfusion (NMP). Dynamic preservation methods utilise the circulation of perfusion solution through the kidney in hypothermia or normothermia and provide a higher benefit for targeted drug delivery. In HMP, the cellular metabolic rate drops to 10% of the metabolic rate at physiological temperature. Moreover, dynamic conditions protect the endothelium. The challenge is to use a drug whose efficacy can be achieved at low temperatures. In NMP there are better conditions for cellular interactions and binding to target sites than in hypothermia. In addition, gene therapies, NPs targeting ischaemia–reperfusion injury or drug delivery can be used during HMP and NMP [140]. 

NMP utilises extracorporeal membrane oxygenation for kidney regeneration before transplantation. It provides a platform for the delivery of drugs, nutrients, gases, mesenchymal stromal cells, gene therapies and new NP-based technologies. It eliminates the problem of delivering drugs to a specific organ and reduces their harmful systemic effects [141]. 

Antibody-conjugated NP drug carriers can be targeted primarily to renal vascular during NMP. NPs targeted on CD31 can increase the level of the NPs in tissues with good blood perfusion. Thanks to NMP, it is possible to deliver drugs into a specific organ, especially ECs [139]. DiRito et al. [142] hypothesised that NPs often accumulate in the microvascular plugs due to decreased perfusion mainly in the renal cortex. In the study performed by the authors on 39 human kidneys, storage in low temperatures induced accumulation of fibrinogen in microvascular and led to the creation of plugs. The plugs can be avoided by using plasminogen and tissue plasminogen activators during NMP.

NPs can be used to assess graft function during normothermic machine perfusion. Woud et al. [11], from their analysis of NPs, including donor-derived extracellular vesicles (EVs) in perfusion fluid, postulate to assess kidney status before transplantation. EVs may reflect the condition of the tissues from which they are derived. They end up in blood or urine and can also help assess organ function after transplantation. The study showed that NPs containing EVs released from kidneys can indicate transplant quality and that analysis of perfusion fluid can assess kidney quality before transplantation. 

Currently, the gold standard for assessing renal function is a histological evaluation of biopsy material. It is an expensive procedure and has limited indications due to its invasiveness. Patients with renal disease and after transplantation require routine assessment of renal function. Feng et al. [143] conducted a study to develop a model to assess kidney function and damage using surface-enhanced Raman spectroscopy (SERS). SERS spectra based on silver NPs were measured in urine. The study showed many dominant vibrational bands due to the strong interaction between urine substances and silver colloids. The urine components were strongly adsorbed on the surface of the silver NPs, and Raman scattering in highly localised optical fields resulted in strong intensity enhancement. The described method is very sensitive to small structural changes. The results showed that SERS spectral analysis of urine could be a method for rapid assessment of kidney function.

Qualification of patients for transplantation is challenging, especially for donors who had AKI and died. Raman analyses often use principal component analysis (PCA) in which data are not labelled and has low sensitivity. Analysis of urine using surface-enhanced Raman spectroscopy is based on applying iodide-modified silver NPs (Ag-I NPs). Using NP in this analysis improves SERS spectra quality and protects the protein against denaturation. SERS analysis is a non-invasive method with great sensitivity (almost 90%) and allows for early assessment of the transplant outcomes from dead donors [144].

Highly specific and sensitive biomarkers are needed to monitor kidney transplant function continuously. Many studies show a relationship between exosomal miRNA, which are endogenous NPs, and renal function. Chen et al. [145] aimed to create a circulating exosomal miRNA panel for the non-invasive assessment of renal function after transplantation. The exosomal miRNA panel has advantages, such as non-invasive procedure, miRNA stability and low susceptibility to external factors, and sensitivity in distinguishing chronic graft dysfunction from normal function. The researchers demonstrated a strong correlation of miR-21, miR-210 and miR4639 in plasma exosomes with eGFR. Furthermore, the diagnostic value of a panel containing miR-21, miR-210 and miR-4639 was significantly better than single and dual indicators. Thus, the utility of an exosomal miRNA panel for non-invasive monitoring of renal allograft function after transplantation was demonstrated. 

### 5.3. Monitoring Patients after KTx

**Figure 4:**
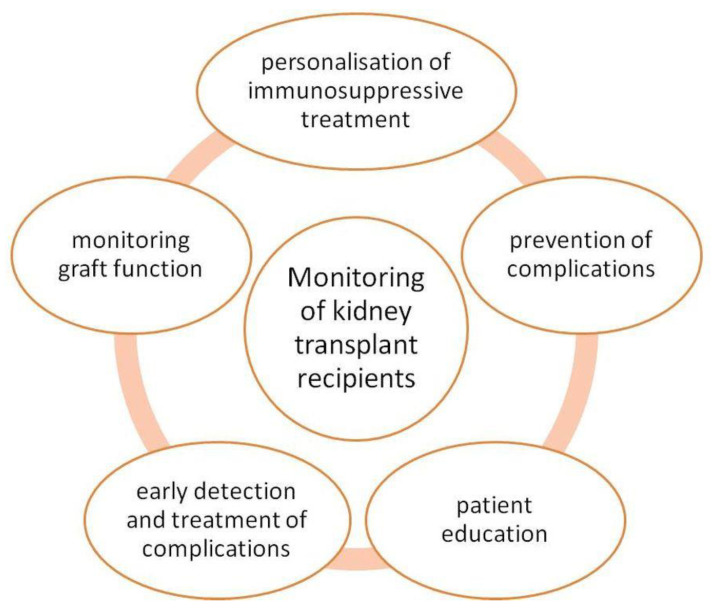
Aspects of transplant recipients monitoring.

The Kidney Disease: Improving Global Outcomes (KDIGO) Transplant Work Group recommends maintaining immunosuppressive therapy for 8–16 weeks after KTx on the condition of no rejection. The KDIGO 2009 recommendations state that tacrolimus should be the first-choice drug to prevent rejection. It reduces the incidence of AR in the first year after KTx. Calcineurin inhibitors (CI) should be started before or during KTx. If acute cellular rejection occurs corticosteroids are recommended as initial treatment [146,147]. KTx recipients should be monitored after surgery [148]. Figure 4 shows the main aspects of the KTx recipient care.

The KDIGO recommends renal biopsy if AR is suspected [147]. In the early period after KTx, it is important to maintain a continuous and undisturbed blood supply to the transplanted kidney. Early complications after KTx are often of vascular origin [149]. Carvalho et al. [150] evaluated surgical complications after KTx. Among 3102 procedures, the most common complications were urinary (n = 184) and vascular (n = 140). Urinary complications included mainly ureteral obstruction and urinary fistula, and vascular difficulties concerned mainly arterial thrombosis and venous thrombosis. This suggests that imaging-based monitoring of these patients should be helpful. It offers the possibility of continuously monitoring transplant recipients. In this case, Doppler ultrasound (DUS) and near-infrared spectroscopy (NIRS) can be used. Both methods are non-invasive. A limitation of DUS is the lack of possibility to monitor perfusion in the critical early post-transplantation period, and NIRS measures only tissue oxygenation [151,152,153]. Non-invasive methods of monitoring early graft function include measuring of serum donor-derived, cell-free DNA, whose potential applications have been described [134]. Non-invasive methods of monitoring are still investigated by many researchers [154]. 

After KTx there is renal ischemia–reperfusion injury (IRI) in which macrophages are involved. In acute kidney IRI macrophages play a significant role. These processes can lead to kidney rejection during which macrophages accumulate and proliferate in the kidney [155]. Chae et al. [127] conducted a study on 18 rats after KTx to evaluate the feasibility of MR imaging of SPIO-labelled macrophages in vivo. The macrophages were incubated with SPIO, and then it was administered intravenously 2 or 5 days after KTx. In the group that received SPIO-labelled macrophages 5 days after KTx, there was increased signal intensity compared to the first group. This study demonstrated the utility of nanotechnology in monitoring potential kidney allograft rejection and better understanding the role of macrophages in graft rejection. 

Potentially, SPIO-enhanced MRI imaging could be useful in imaging the immune response after KTx. Further research in this direction is needed [155]. In the non-invasive diagnosis of rejection after KTx, ferumoxtran-10 can be helpful. This USPIO NP is not filtered by the glomerulus [41]. Ferumoxtran-10 is an iron oxide NP coated with dextran, which is biodegradable, with a maximum size of 50 nm. It is used as an MRI agent [156].

Aghighi et al. [157] studied a group of 26 patients under 26 to test the usefulness of USPIO-enhanced magnetic resonance imaging in detecting kidney allograft rejection. They used USPIO-ferumoxytol injected intravenously. Allografts with AR had prolonged T2* values on ferumoxytol-enhanced MRI images compared to the group without rejection. Subsequent MRI images and histopathology of the kidney with AR showed no significant retention of ferumoxytol. Yang et al. [158] evaluated USPIO on normal and transplanted rat kidneys. Renal graft perfusion was measured and correlated with histopathological findings. The rats with transplanted kidneys had a lower maximum signal decrease and lower wash-in slope. In KTx rats there was acute vascular rejection. The authors indicated that the dose of 6.0 mg Fe/kg is optimal for imaging renal perfusion and fast in rat kidney wash-out. Similar studies using USPIO and MR imaging were conducted on humans. Hauger et al. [159] evaluated whether USPIO can be useful for detecting macrophage infiltration in native and transplanted kidneys. The study involved an injection of 1.7–2.6 mg Fe/kg. It showed that MR imaging with USPIO enhanced can be helpful in the detection of renal inflammation due to the capacity to depict infiltration macrophages. The patients with >5 macrophages/mm^2^ showed a cortical signal loss on T2*-weighted images.

Cytotoxic CD8+ T cells are released during acute cellular rejection after skin transplantation. These cells infiltrate the allograft during rejection and release serine protease, which is granzyme B. It could be a potential way for research in the area of KTx [10]. Halloran et al. [160] studied the role of granzymes B in cytotoxic in T-cell-mediated kidney transplant rejection in mice. It appeared that renal allograft rejection was accompanied by a massively elevated and persistent expression of perforin and granzymes A and B. Still, this increase did not correlate with the development of tubulitis or arteritis. This study showed that the mentioned rejection is not mediated by granzymes B but is associated with this protease. Granule-associated T cell cytotoxic mechanisms do not cause allograft rejection by T cells.

**Figure 5:**
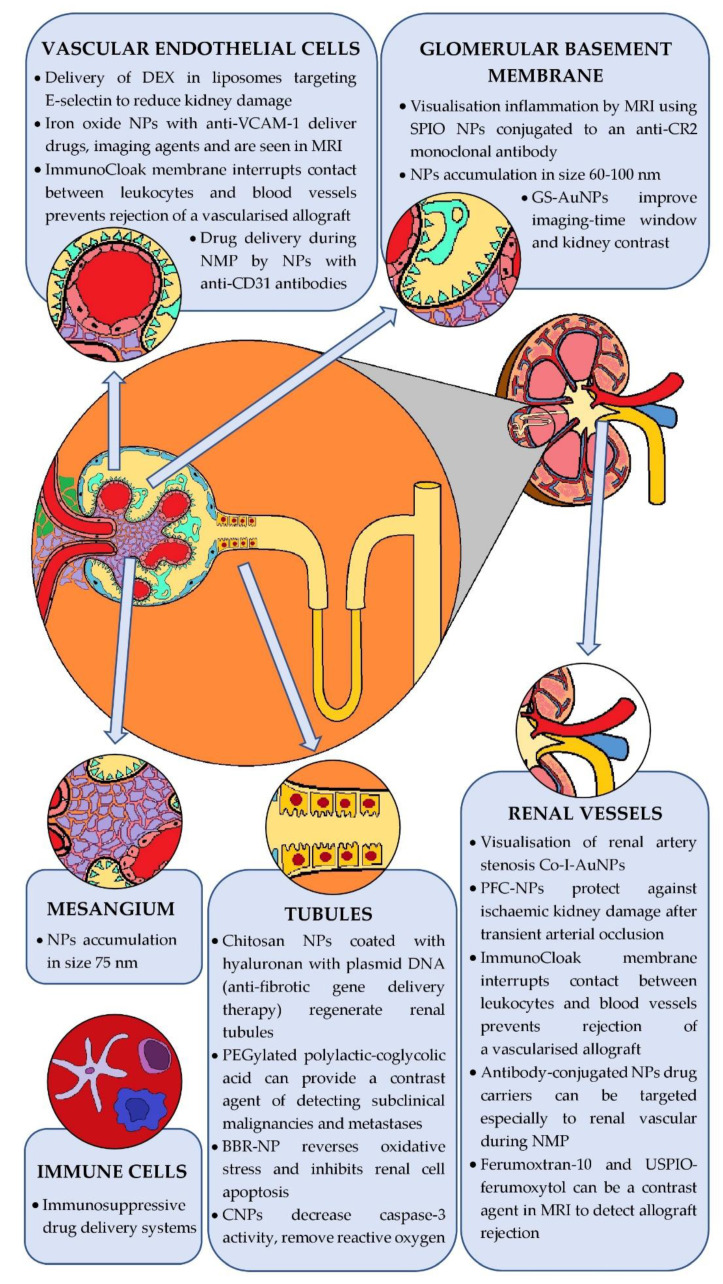
Different target sites for nanoparticles in kidney diseases.

Figure 5 summarises the use of nanoparticles at different target sites in kidney disease diagnostics and treatment.

### 5.4. Limitations of Nanoparticles in Nephrology

While modest information can be found on the environmental toxicity of NPs [161], the renal toxicity of NPs is not very well documented. It is known that NPs interact with biomolecules to increase their bioavailability and half-life. Moreover, they are involved in very complex interactions, making toxicity assessment difficult [162]. The nephrotoxicity of nanoparticles is not well studied. Metabolites of nanoparticles pass through the kidney. It is not known whether the use of nanoparticles targeted to organs other than the kidneys have a negative effect on the kidneys. In addition, the accumulation of nanoparticles and their metabolites in the kidney is not studied. 

Further studies on toxicity, accumulation effects, longer half-life and interactions with other endogenous and exogenous molecules are required. The use of NPs in medicine is an emerging area that has not been explored thoroughly yet. Few clinical trials have been conducted thus far, and most of them involved animals. Therefore, it will still take time to undertake studies that will dispel existing doubts. 

## 6. Conclusions and Future Directions

Our work summarises the findings to date of nanomedicine in nephrology, but also in kidney transplantation. NPs are a very promising form of kidney disease therapy and diagnostics. Non-invasive markers are expected in nephrological and kidney transplant societies to avoid invasive biopsy of one’s own or transplanted kidney. NPs should allow for earlier diagnosis of kidney injuries, which should help to avoid dialysis or prolong transplant survival. 

NPs provide valuable information by targeting specific diseased sites, which is superior to other diagnostic and therapeutic methods. Studies to date report that NPs show no toxicity, display durability and are relatively easy to produce. The broad potential of NP designs represents the future of kidney transplantation, for which selective action on the immune system, assessment of transplanted organ function and damage detection are critical. However, data on the toxicity and efficacy of NPs are insufficient and further studies are required.

## Figures and Tables

**Table 1:** Clinical trials concerning nanoparticles in kidney diseases downloaded from the ClinicalTrials.gov database (https://clinicaltrials.gov/, accessed on 30 September 2021).

NCT Number	Title	Condition	Actual Enrolment ^1^	Recruitment Status	Location	Age of Participants ^2^
NCT05045872	The Accuracy and Safety of Renal Artery Contrast-enhanced Magnetic Resonance Imaging with Polysaccharide Superparamagnetic Iron Oxide Nanoparticle	Chronic Kidney Diseases	40 ^3^	Not yet recruiting	China	Adult, Older Adult
NCT04277377	Nanoparticle for DSA Removal	Kidney Failure, Presence of Donor Specific Antibodies	100 ^3^	Not yet recruiting	Switzerland	Adult, Older Adult
NCT02646319	Nanoparticle Albumin-Bound Rapamycin in Treating Patients with Advanced Cancer with mTOR Mutations	Advanced Cancers ^4^	2	Completed	United States	Adult, Older Adult
NCT04260360	Trial of NanoDoce Intratumoral Injection in Renal Cell Carcinoma	Renal Cell Carcinoma, Kidney Cancer, Adenocarcinoma of Kidney, Adenocarcinoma, Renal, Renal Cell Cancer	0	Withdrawn (Not initiated)	No data	Adult, Older Adult
NCT02006108	Imaging Kidney Transplant Rejection Using Ferumoxytol-Enhanced Magnetic Resonance	Renal Transplant Rejection	21	Completed	United States	Child, Adult
NCT03961698	Evaluation of IPI-549 Combined with Front-line Treatments in Pts. With Triple-Negative Breast Cancer or Renal Cell Carcinoma (MARIO-3) (MARIO-3)	Breast Cancer, Renal Cell Carcinoma	90 ^3^	Recruiting	United States	Adult, Older Adult
NCT00499291	Paclitaxel Albumin-Stabilized Nanoparticle Formulation in Treating Patients with Advanced or Refractory Solid Tumors	Cancer	0	Withdrawn	No data	Adult, Older Adult
NCT02626663	The Role of Microparticles as a Biomarker	Atypical Haemolytic Uremic Syndrome, Thrombotic Thrombocytopenic Purpura, Microparticles, Microangiopathic Haemolytic Anaemia	0	Withdrawn (PI left the University, sponsor pulled funding)	United States	Adult, Older Adult
NCT03678883	9-ING-41 in Patients with Advanced Cancers	Human malignancies ^5^	350 ^3^	Recruiting	United States, Netherlands, Spain	Adult, Older Adult
NCT00689065	Safety Study of CALAA-01 to Treat Solid Tumor Cancers	Cancer, Solid Tumour	24	Terminated	United States	Adult, Older Adult

^1^ Number of participants. ^2^ Child—birth-17 years old; adult—18–64 years old; older adult—more than 65 years old; ^3^ Estimated Enrolment; ^4^ Advanced Malignant Neoplasm, Cervical Squamous Cell Carcinoma, Endometrial Carcinoma, Malignant Uterine Neoplasm, Recurrent Bladder Carcinoma, Recurrent Breast Carcinoma Recurrent Cervical Carcinoma Recurrent Head and Neck Carcinoma Recurrent Malignant Neoplasm Recurrent Ovarian Carcinoma Recurrent Prostate Carcinoma Recurrent Renal Cell Carcinoma Solid Neoplasm Stage III Bladder Cancer Stage III Prostate Cancer Stage III Renal Cell Cancer Stage IIIA Breast Cancer Stage IIIA Cervical Cancer Stage IIIA Ovarian Cancer Stage IIIB Breast Cancer Stage IIIB Cervical Cancer Stage IIIB Ovarian Cancer Stage IIIC Breast Cancer Stage IIIC Ovarian Cancer Stage IV Breast Cancer Stage IV Ovarian Cancer Stage IV Prostate Cancer Stage IV Renal Cell Cancer Stage IVA Bladder Cancer Stage IVA Cervical Cancer Stage IVB Bladder Cancer Stage IVB Cervical Cancer; ^5^ Cancer, Pancreatic Cancer, Sarcoma, Renal Cancer, Refractory Cancer, Refractory Neoplasm, Refractory Non-Hodgkin Lymphoma, Pancreatic Adenocarcinoma, Resistant Cancer, Neoplasm Metastasis, Neoplasm of Bone, Neoplasm, Breast, Neoplasm of Lung, Neoplasms, Colorectal, Neoplasms Pancreatic, Malignant Glioma, Malignancies, Malignancies Multiple, Bone Metastases, Bone Neoplasm, Bone Cancer, Pancreas Cancer, Pancreatic Neoplasms, Breast Neoplasms, Acute T Cell Leukaemia Lymphoma.

**Table 2:** Non-clinical studies of nanoparticles in kidney diseases and kidney transplantation.

Nanoparticles	Type	Role	Mechanism of Action	Type of Condition/ Pathology	References
Gold NPs with anti-collage-I antibody	Nanocarriers with molecules on the surface that interact with target cells	Diagnostics	CT-imaging contrast, increased retention of gold NPs, good safety profile.	Renal fibrosis	[113]
SPIO NPs conjugated to an anti-CR2 monoclonal antibody	SPIO/Nanocarriers with drugs	Therapeutic and diagnostic	Reduced inflammation -targeted drug delivery (e.g., dexamethasone) to glomerular endothelial cells, visualization of inflammation by MRI	Glomerulonephritis	[114]
Iron oxide NPs conjugated to anti-VCAM-1 antibody	SPIO/Nanocarriers with drugs	Therapeutic and diagnostic	MRI imaging of the kidney and targeted drug delivery to ischemic tissue (with increased VCAM-1 expression)	AKI (ischemia-reperfusion injury)	[114]
Mesoscale NPs	Nanocarriers with drugs	Therapeutic	Drug delivery with 5–7- fold higher efficacy in the kidney (tubular epithelium) than in other organs, safety profile.	Unspecified (various kidney pathologies)	[115]
Glutathione-coated luminescent gold NPs	Renal-clearable luminescent metal NPs	Diagnostic	Improved imaging-time window and kidney contrast compared to conventional dyes— superior assessment of renal function	Unspecified (various kidney pathologies)	[116]
PEGylated polylactic-coglycolic acid NPs with contrast/drug	Nanocarriers with molecules on the surface that interact with target cells	Therapeutic and diagnostic	Selective interaction with proximal tubule epithelial cells and renal clear cell carcinoma cells—new possibility of imaging a kidney tumour	Renal clear cell carcinoma	[117]
Zn/CuO NPs	Nanocarriers with cytotoxic agents	Therapeutic	Excellent inhibition of renal cancer cells- cytotoxic effect of zinc ions without affecting healthy cells	Renal tumours	[118]
Liposomes with immunosuppressive drugs	Nanocarriers with immunosuppressive drugs	Therapeutic	Targeted drug delivery with lower toxicity than immunosuppressive drugs alone	Graft rejection	[119]
Thrombin-targeted PFC-NP	Nanocarriers with anticoagulants	Therapeutic	Antithrombin activity- protection of the transplanted kidney after a period of ischaemia	Transient arterial occlusion after KTx	[120]
NP carrier of mycophenolate mofetil	Nanocarriers with immunosuppressive drugs	Therapeutic	Direct delivery of immunosuppressive drugs to the organ at the time of transplantation reduces intra- graft inflammation and alloimmune activation at a critical time	Graft rejection	[121]
NP carrier of tacrolimus	Nanocarriers with immunosuppressive drugs	Therapeutic	Inhibition of interleukin-2 production, suppression of T-cell activation with good safety profile	Graft rejection	[122]
Rapamycin and ovalbumin peptide loaded porous silicon NPs	Nanocarriers with immunosuppressive drugs	Therapeutics	Up-regulation of regulatory T-cells	Graft rejection	[123]
Immunocloaking membrane covering luminal surfaces of allograft vessels	Nanomaterial	Therapeutics	Interrupted antigen presentation, disrupted diapedesis and inhibited early T-cell activation	Graft rejection	[124]
Berberine NPs	Nanocarriers with antioxidative agents	Therapeutic	Reduced expression of oxidative and mitochondrial stress pathway proteins	AKI (ischemia-reperfusion injury)	[125]
CNPs	Nanocarriers with antioxidative agents	Therapeutic	Decreased caspase-3 activity, reduced level of reactive oxygen species	AKI (ischemia-reperfusion injury)	[126]
SPIO-labelled macrophages	SPIO	Diagnostic	Increased signal in MRI—new possibility of monitoring of macrophages accumulation in kidney and potential kidney allograft rejection	Renal ischemia (reperfusion injury after KTx)	[127]

Acute kidney injury (AKI); Anti-vascular cell adhesion molecule-1 (anti-VCAM-1); Cerium oxide nanoparticles (CNPs); Computed tomography (CT); Kidney transplantation (KTx); Magnetic resonance imaging (MRI); Nanoparticle (NP); Perfluorocarbon nanoparticle (PFC-NP); Superparamagnetic iron oxide (SPIO).

## Data Availability

No new data were created or analysed in this study. Data sharing is not applicable to this article.

## Abbreviations

Acute kidney injury (AKI); acute rejection (AR); anti-vascular cell adhesion molecule-1 (anti-VCAM-1); Berberine NPs (BBR-NP); Calcineurin inhibitors (CI); central nervous system (CNS); Cerium oxide NPs (CNPs); computed tomography (CT); coronavirus disease 2019 (COVID-19); Delayed graft function (DGF); dimensions (D); Doppler ultrasound (DUS); doxorubicin (DOX); extracellular vesicles (EVs); Focal segmental glomerulosclerosis (FSGS); glutathione-coated luminescent gold NPs (GS-AuNPs); gold NPs with anti-collagen-I antibody (Co-I-AuNPs); Hepatitis B Virus (HBV); Hepatitis C Virus (HCV); human epithelial cells (HK-2); Human Immunodeficiency Virus (HIV); Human serum albumin (HSA); hypothermic machine perfusion (HMP); immunosuppressive agents (ISAs); institutional review board (IRB); iodide-modified silver NPs (Ag-I NPs); ischemia-reperfusion injury (IRI); kidney clearance kinetics (KCK); kidney transplantation (KTx); kinesin spindle protein (KSP); layer-by-layer (LbL); magnetic resonance imaging (MRI); mesoscale NPs (MNPs); Microbubbles (MBs); mycophenolate mofetil (MMF); Nanomaterials (NMs); nanometers (nm); nanoparticle albumin-bound paclitaxel (nab-Ptx); Nanoparticles (NPs); nanostructure lipid carriers (NLCs); near-infrared spectroscopy (NIRS); normothermic machine perfusion (NMP); NPs drug-eluting stents (NP-DES); Paclitaxel (Ptx); perfluorocarbon NP (PFC-NP); Porous silicon NPs (pSiNPs); principal component analysis (PCA); quantum dots (QDs); respiratory syncytial virus (RSV); severe acute respiratory syndrome coronavirus 2 (SARS-CoV-2); small interfering RNA (siRNA); solid lipid NPs (SLNs); superparamagnetic iron oxide (SPIO); surface-enhanced Raman spectroscopy (SERS); Systematic Review and Meta-Analysis (PRISMA); tacrolimus (TAC); Ultra-small gold nanoclusters (AuNCs); Ultra-small particles of iron oxide (USPIO); Vascular cell adhesion molecule-1 (VCAM-1); vascular endothelial cells (ECs); Vascular endothelial growth factor (VEGF).

## References

[1] Gatoo M.A., Naseem S., Arfat M.Y., Mahmood Dar A., Qasim K., Zubair S. (2014). Physicochemical Properties of Nanomaterials: Implication in Associated Toxic Manifestations. BioMed Res. Int..

[2] Shatrohan Lal R.K. (2014). Synthesis of Organic Nanoparticles and Their Applications in Drug Delivery and Food Nanotechnology: A Review. J. Nanomater. Mol. Nanotechnol..

[3] Madkour L.H. (2019). Introduction to Nanotechnology (NT) and Nanomaterials (NMs). Nanoelectronic Materials.

[4] Kumar Teli M., Mutalik S., Rajanikant G.K. (2010). Nanotechnology and Nanomedicine: Going Small Means Aiming Big. Curr. Pharm. Des..

[5] Nowack B., Bucheli T.D. (2007). Occurrence, Behavior and Effects of Nanoparticles in the Environment. Environ. Pollut..

[6] Saleh T.A. (2020). Nanomaterials: Classification, Properties, and Environmental Toxicities. Environ. Technol. Innov..

[7] Ma Y., Cai F., Li Y., Chen J., Han F., Lin W. (2020). A Review of the Application of Nanoparticles in the Diagnosis and Treatment of Chronic Kidney Disease. Bioact. Mater..

[8] Bordea I.R., Candrea S., Alexescu G.T., Bran S., Băciuț M., Băciuț G., Lucaciu O., Dinu C.M., Todea D.A. (2020). Nano-Hydroxyapatite Use in Dentistry: A Systematic Review. Drug Metab. Rev..

[9] Huang Y., Wang J., Jiang K., Chung E.J. (2021). Improving Kidney Targeting: The Influence of Nanoparticle Physicochemical Properties on Kidney Interactions. J. Control. Release.

[10] Mac Q.D., Mathews D.V., Kahla J.A., Stoffers C.M., Delmas O.M., Holt B.A., Adams A.B., Kwong G.A. (2019). Non-Invasive Early Detection of Acute Transplant Rejection via Nanosensors of Granzyme B Activity. Nat. Biomed. Eng..

[11] Woud W.W., Merino A., Hoogduijn M.J., Boer K., van den Hoogen M.W.F., Baan C.C., Minnee R.C. (2019). Nanoparticle Release by Extended Criteria Donor Kidneys During Normothermic Machine Perfusion. Transplantation.

[12] Asha A.B., Narain R. (2020). Nanomaterials Properties. Polymer Science and Nanotechnology.

[13] Trotta F., Mele A., Trotta F., Mele A. (2019). Nanomaterials: Classification and Properties. Nanosponges.

[14] Christian P., Von der Kammer F., Baalousha M., Hofmann T. (2008). Nanoparticles: Structure, Properties, Preparation and Behaviour in Environmental Media. Ecotoxicology.

[15] Turner M., Golovko V.B., Vaughan O.P.H., Abdulkin P., Berenguer-Murcia A., Tikhov M.S., Johnson B.F.G., Lambert R.M. (2008). Selective Oxidation with Dioxygen by Gold Nanoparticle Catalysts Derived from 55-Atom Clusters. Nature.

[16] Sau T.K., Pal A., Pal T. (2001). Size Regime Dependent Catalysis by Gold Nanoparticles for the Reduction of Eosin. J. Phys. Chem. B.

[17] Eustis S., El-Sayed M.A. (2006). Why Gold Nanoparticles Are More Precious than Pretty Gold: Noble Metal Surface Plasmon Resonance and Its Enhancement of the Radiative and Nonradiative Properties of Nanocrystals of Different Shapes. Chem. Soc. Rev..

[18] Clogston J.D., Patri A.K., McNeil S.E. (2011). Zeta Potential Measurement. Characterization of Nanoparticles Intended for Drug Delivery.

[19] Kumar N., Sinha Ray S., Sinha Ray S. (2018). Synthesis and Functionalization of Nanomaterials. Processing of Polymer-based Nanocomposites.

[20] Sanvicens N., Marco M.P. (2008). Multifunctional Nanoparticles—Properties and Prospects for Their Use in Human Medicine. Trends Biotechnol..

[21] Kim D., Shin K., Kwon S.G., Hyeon T. (2018). Synthesis and Biomedical Applications of Multifunctional Nanoparticles. Adv. Mater..

[22] Khalid K., Tan X., Mohd Zaid H.F., Tao Y., Lye Chew C., Chu D.-T., Lam M.K., Ho Y.-C., Lim J.W., Chin Wei L. (2020). Advanced in Developmental Organic and Inorganic Nanomaterial: A Review. Bioengineered.

[23] Subbiah R., Veerapandian M., Yun K.S. (2010). Nanoparticles: Functionalization and Multifunctional Applications in Biomedical Sciences. Curr. Med. Chem..

[24] Kumar N., Sinha Ray S., Ngila J.C. (2017). Ionic Liquid-Assisted Synthesis of Ag/Ag_2_ Te Nanocrystals via a Hydrothermal Route for Enhanced Photocatalytic Performance. New J. Chem..

[25] Gusain R., Singhal N., Singh R., Kumar U., Khatri O.P. (2016). Ionic-Liquid-Functionalized Copper Oxide Nanorods for Photocatalytic Splitting of Water. ChemPlusChem.

[26] Anselmo A.C., Mitragotri S. (2019). Nanoparticles in the Clinic: An Update. Bioeng. Transl. Med..

[27] Cherukula K., Manickavasagam Lekshmi K., Uthaman S., Cho K., Cho C.-S., Park I.-K. (2016). Multifunctional Inorganic Nanoparticles: Recent Progress in Thermal Therapy and Imaging. Nanomaterials.

[28] Busquets M.A., Estelrich J., Sánchez-Martín M.J. (2015). Nanoparticles in Magnetic Resonance Imaging: From Simple to Dual Contrast Agents. Int. J. Nanomed..

[29] Das S., Kotcherlakota R., Patra C.R., Shukla A.K. (2019). Noninvasive Imaging Techniques of Metal Nanoparticles and Their Future Diagnostic Applications. Medical Imaging Methods.

[30] Karakatsanis A., Christiansen P.M., Fischer L., Hedin C., Pistioli L., Sund M., Rasmussen N.R., Jørnsgård H., Tegnelius D., Eriksson S. (2016). The Nordic SentiMag Trial: A Comparison of Super Paramagnetic Iron Oxide (SPIO) Nanoparticles versus Tc99 and Patent Blue in the Detection of Sentinel Node (SN) in Patients with Breast Cancer and a Meta-Analysis of Earlier Studies. Breast Cancer Res. Treat..

[31] Taruno K., Kurita T., Kuwahata A., Yanagihara K., Enokido K., Katayose Y., Nakamura S., Takei H., Sekino M., Kusakabe M. (2019). Multicenter Clinical Trial on Sentinel Lymph Node Biopsy Using Superparamagnetic Iron Oxide Nanoparticles and a Novel Handheld Magnetic Probe. J. Surg. Oncol..

[32] Rosen J.E., Yoffe S., Meerasa A. (2011). Nanotechnology and Diagnostic Imaging: New Advances in Contrast Agent Technology. J. Nanomedic. Nanotechnol..

[33] Zhao M.-X., Zeng E.-Z. (2015). Application of Functional Quantum Dot Nanoparticles as Fluorescence Probes in Cell Labeling and Tumor Diagnostic Imaging. Nanoscale Res. Lett..

[34] Tavernaro I., Cavelius C., Peuschel H., Kraegeloh A. (2017). Bright Fluorescent Silica-Nanoparticle Probes for High-Resolution STED and Confocal Microscopy. Beilstein J. Nanotechnol..

[35] Kosareva A., Abou-Elkacem L., Chowdhury S., Lindner J.R., Kaufmann B.A. (2020). Seeing the Invisible—Ultrasound Molecular Imaging. Ultrasound Med. Biol..

[36] Perera R., de Leon A., Wang X., Wang Y., Ramamurthy G., Peiris P., Abenojar E., Basilion J.P., Exner A.A. (2020). Real Time Ultrasound Molecular Imaging of Prostate Cancer with PSMA-Targeted Nanobubbles. Nanomed. Nanotechnol. Biol. Med..

[37] Gao Y., Hernandez C., Yuan H.-X., Lilly J., Kota P., Zhou H., Wu H., Exner A.A. (2017). Ultrasound Molecular Imaging of Ovarian Cancer with CA-125 Targeted Nanobubble Contrast Agents. Nanomed. Nanotechnol. Biol. Med..

[38] Ishigami K., Nishie A., Nakayama T., Asayama Y., Kakihara D., Fujita N., Ushijima Y., Okamoto D., Ohtsuka T., Mori Y. (2019). Superparamagnetic Iron-Oxide-Enhanced Diffusion-Weighted Magnetic Resonance Imaging for the Diagnosis of Intrapancreatic Accessory Spleen. Abdom. Radiol..

[39] Kumano S., Murakami T., Kim T., Hori M., Okada A., Sugiura T., Noguchi Y., Kawata S., Tomoda K., Nakamura H. (2003). Using Superparamagnetic Iron Oxide–Enhanced MRI to Differentiate Metastatic Hepatic Tumors and Nonsolid Benign Lesions. Am. J. Roentgenol..

[40] Umetsu M., Goto H., Nakamura Y., Ota H., Shimizu T., Hashimoto M., Akamatsu D., Kamei T. (2020). Detection of Macrophage Localization in the Abdominal Aortic Aneurysm Wall Using Ex Vivo Superparamagnetic Iron Oxide–Enhanced Magnetic Resonance Imaging. Ann. Vasc. Surg..

[41] Sharfuddin A. (2014). Renal Relevant Radiology: Imaging in Kidney Transplantation. Clin. J. Am. Soc. Nephrol..

[42] Bejarano J., Navarro-Marquez M., Morales-Zavala F., Morales J.O., Garcia-Carvajal I., Araya-Fuentes E., Flores Y., Verdejo H.E., Castro P.F., Lavandero S. (2018). Nanoparticles for Diagnosis and Therapy of Atherosclerosis and Myocardial Infarction: Evolution toward Prospective Theranostic Approaches. Theranostics.

[43] Kao C.-W., Wu P.-T., Liao M.-Y., Chung I.-J., Yang K.-C., Tseng W.-Y., Yu J. (2018). Magnetic Nanoparticles Conjugated with Peptides Derived from Monocyte Chemoattractant Protein-1 as a Tool for Targeting Atherosclerosis. Pharmaceutics.

[44] Lagan J., Naish J.H., Simpson K., Zi M., Cartwright E.J., Foden P., Morris J., Clark D., Birchall L., Caldwell J. (2021). Substrate for the Myocardial Inflammation–Heart Failure Hypothesis Identified Using Novel USPIO Methodology. JACC Cardiovasc. Imaging.

[45] Kang J., Tahir A., Wang H., Chang J. (2021). Applications of Nanotechnology in Virus Detection, Tracking, and Infection Mechanisms. WIREs Nanomed. Nanobiotechnol..

[46] Valizadeh A., Mikaeili H., Samiei M., Farkhani S.M., Zarghami N., kouhi M., Akbarzadeh A., Davaran S. (2012). Quantum Dots: Synthesis, Bioapplications, and Toxicity. Nanoscale Res. Lett..

[47] Draz M.S., Shafiee H. (2018). Applications of Gold Nanoparticles in Virus Detection. Theranostics.

[48] Aithal S., Mishriki S., Gupta R., Sahu R.P., Botos G., Tanvir S., Hanson R.W., Puri I.K. (2022). SARS-CoV-2 Detection with Aptamer-Functionalized Gold Nanoparticles. Talanta.

[49] Wang J., Drelich A.J., Hopkins C.M., Mecozzi S., Li L., Kwon G., Hong S. (2021). Gold Nanoparticles in Virus Detection: Recent Advances and Potential Considerations for SARS-CoV-2 Testing Development. WIREs Nanomed. Nanobiotechnol..

[50] Pramanik A., Gao Y., Patibandla S., Mitra D., McCandless M.G., Fassero L.A., Gates K., Tandon R., Chandra Ray P. (2021). The Rapid Diagnosis and Effective Inhibition of Coronavirus Using Spike Antibody Attached Gold Nanoparticles. Nanoscale Adv..

[51] Lew T.T.S., Aung K.M.M., Ow S.Y., Amrun S.N., Sutarlie L., Ng L.F.P., Su X. (2021). Epitope-Functionalized Gold Nanoparticles for Rapid and Selective Detection of SARS-CoV-2 IgG Antibodies. ACS Nano.

[52] Saxena A. (2020). Biotechnology Business-Concept to Delivery.

[53] Wang H., Cheng G., Du Y., Ye L., Chen W., Zhang L., Wang T., Tian J., Fu F. (2013). Hypersensitivity Reaction Studies of a Polyethoxylated Castor Oil-Free, Liposome-Based Alternative Paclitaxel Formulation. Mol. Med. Rep..

[54] Gardner E.R., Dahut W.L., Scripture C.D., Jones J., Aragon-Ching J.B., Desai N., Hawkins M.J., Sparreboom A., Figg W.D. (2008). Randomized Crossover Pharmacokinetic Study of Solvent-Based Paclitaxel and Nab-Paclitaxel. Clin. Cancer Res..

[55] Untch M., Jackisch C., Schneeweiss A., Schmatloch S., Aktas B., Denkert C., Schem C., Wiebringhaus H., Kümmel S., Warm M. (2019). NAB-Paclitaxel Improves Disease-Free Survival in Early Breast Cancer: GBG 69–GeparSepto. JCO.

[56] Mahtani R.L., Parisi M., Glück S., Ni Q., Park S., Pelletier C., Faria C., Braiteh F. (2018). Comparative Effectiveness of Early-Line Nab-Paclitaxel vs. Paclitaxel in Patients with Metastatic Breast Cancer: A US Community-Based Real-World Analysis. CMAR.

[57] Goldstein D., El-Maraghi R.H., Hammel P., Heinemann V., Kunzmann V., Sastre J., Scheithauer W., Siena S., Tabernero J., Teixeira L. (2015). Nab-Paclitaxel Plus Gemcitabine for Metastatic Pancreatic Cancer: Long-Term Survival from a Phase III Trial. JNCI J. Natl. Cancer Inst..

[58] Weiss J., Gilbert J., Deal A.M., Weissler M., Hilliard C., Chera B., Murphy B., Hackman T., Liao J.J., Grilley Olson J. (2018). Induction Chemotherapy with Carboplatin, Nab-Paclitaxel and Cetuximab for at Least N2b Nodal Status or Surgically Unresectable Squamous Cell Carcinoma of the Head and Neck. Oral Oncol..

[59] Mudad R., Patel M.B., Margunato-Debay S., Garofalo D., Lal L.S. (2017). Comparative Effectiveness and Safety of Nab-Paclitaxel plus Carboplatin vs Gemcitabine plus Carboplatin in First-Line Treatment of Advanced Squamous Cell Non-Small Cell Lung Cancer in a US Community Oncology Setting. LCTT.

[60] Tiwari G., Tiwari R., Bannerjee S., Bhati L., Pandey S., Pandey P., Sriwastawa B. (2012). Drug Delivery Systems: An Updated Review. Int. J. Pharm. Investig..

[61] Stylianopoulos T., Jain R.K. (2015). Design Considerations for Nanotherapeutics in Oncology. Nanomed. Nanotechnol. Biol. Med..

[62] Batist G., Ramakrishnan G., Rao C.S., Chandrasekharan A., Gutheil J., Guthrie T., Shah P., Khojasteh A., Nair M.K., Hoelzer K. (2001). Reduced Cardiotoxicity and Preserved Antitumor Efficacy of Liposome-Encapsulated Doxorubicin and Cyclophosphamide Compared with Conventional Doxorubicin and Cyclophosphamide in a Randomized, Multicenter Trial of Metastatic Breast Cancer. JCO.

[63] Valero V., Buzdar A.U., Theriault R.L., Azarnia N., Fonseca G.A., Willey J., Ewer M., Walters R.S., Mackay B., Podoloff D. (1999). Phase II Trial of Liposome-Encapsulated Doxorubicin, Cyclophosphamide, and Fluorouracil as First-Line Therapy in Patients with Metastatic Breast Cancer. JCO.

[64] Kepinska M., Kizek R., Milnerowicz H. (2018). Metallothionein and Superoxide Dismutase—Antioxidative Protein Status in Fullerene-Doxorubicin Delivery to MCF-7 Human Breast Cancer Cells. IJMS.

[65] Lei J., Wang H., Zhu D., Wan Y., Yin L. (2020). Combined Effects of Avasimibe Immunotherapy, Doxorubicin Chemotherapy, and Metal–Organic Frameworks Nanoparticles on Breast Cancer. J. Cell Physiol..

[66] Graziani S.R., Vital C.G., Morikawa A.T., Van Eyll B.M., Fernandes Junior H.J., Kalil Filho R., Maranhão R.C. (2017). Phase II Study of Paclitaxel Associated with Lipid Core Nanoparticles (LDE) as Third-Line Treatment of Patients with Epithelial Ovarian Carcinoma. Med. Oncol..

[67] Kepinska M., Kizek R., Milnerowicz H. (2018). Fullerene as a Doxorubicin Nanotransporter for Targeted Breast Cancer Therapy: Capillary Electrophoresis Analysis. Electrophoresis.

[68] Yu X., Trase I., Ren M., Duval K., Guo X., Chen Z. (2016). Design of Nanoparticle-Based Carriers for Targeted Drug Delivery. J. Nanomater..

[69] Wartlick H., Michaelis K., Balthasar S., Strebhardt K., Kreuter J., Langer K. (2004). Highly Specific HER2-Mediated Cellular Uptake of Antibody-Modified Nanoparticles in Tumour Cells. J. Drug Target..

[70] Ranghar S., Sirohi P., Verma P., Agarwal V. (2013). Nanoparticle-Based Drug Delivery Systems: Promising Approaches against Infections. Braz. Arch. Biol. Technol..

[71] Ruttkay-Nedecky B., Skalickova S., Kepinska M., Cihalova K., Docekalova M., Stankova M., Uhlirova D., Fernandez C., Sochor J., Milnerowicz H. (2019). Development of New Silver Nanoparticles Suitable for Materials with Antimicrobial Properties. J. Nanosci. Nanotechnol..

[72] Li Y., Lin Z., Guo M., Zhao M., Xia Y., Wang C., Xu T., Zhu B. (2018). Inhibition of H1N1 Influenza Virus-Induced Apoptosis by Functionalized Selenium Nanoparticles with Amantadine through ROS-Mediated AKT Signaling Pathways. IJN.

[73] Li Y., Lin Z., Guo M., Xia Y., Zhao M., Wang C., Xu T., Chen T., Zhu B. (2017). Inhibitory Activity of Selenium Nanoparticles Functionalized with Oseltamivir on H1N1 Influenza Virus. IJN.

[74] Dube A., Reynolds J.L., Law W.-C., Maponga C.C., Prasad P.N., Morse G.D. (2014). Multimodal Nanoparticles That Provide Immunomodulation and Intracellular Drug Delivery for Infectious Diseases. Nanomed. Nanotechnol. Biol. Med..

[75] Saraiva C., Praça C., Ferreira R., Santos T., Ferreira L., Bernardino L. (2016). Nanoparticle-Mediated Brain Drug Delivery: Overcoming Blood–Brain Barrier to Treat Neurodegenerative Diseases. J. Control. Release.

[76] Lam F.C., Morton S.W., Wyckoff J., Vu Han T.-L., Hwang M.K., Maffa A., Balkanska-Sinclair E., Yaffe M.B., Floyd S.R., Hammond P.T. (2018). Enhanced Efficacy of Combined Temozolomide and Bromodomain Inhibitor Therapy for Gliomas Using Targeted Nanoparticles. Nat. Commun..

[77] Wang Y., Rajala A., Rajala R. (2015). Lipid Nanoparticles for Ocular Gene Delivery. JFB.

[78] Ostróżka-Cieślik A., Sarecka-Hujar B. (2017). The Use of Nanotechnology in Modern Pharmacotherapy. Multifunctional Systems for Combined Delivery, Biosensing and Diagnostics.

[79] Lin Y.-L., Tsai N.-M., Chen C.-H., Liu Y.-K., Lee C.-J., Chan Y.-L., Wang Y.-S., Chang Y.-C., Lin C.-H., Huang T.-H. (2019). Specific Drug Delivery Efficiently Induced Human Breast Tumor Regression Using a Lipoplex by Non-Covalent Association with Anti-Tumor Antibodies. J. Nanobiotechnol..

[80] Zhang X., Liang T., Ma Q. (2021). Layer-by-Layer Assembled Nano-Drug Delivery Systems for Cancer Treatment. Drug Deliv..

[81] Theek B., Rizzo L.Y., Ehling J., Kiessling F., Lammers T. (2014). The Theranostic Path to Personalized Nanomedicine. Clin. Transl. Imaging.

[82] Baetke S.C., Lammers T., Kiessling F. (2015). Applications of Nanoparticles for Diagnosis and Therapy of Cancer. BJR.

[83] Sharma A., Goyal A.K., Rath G. (2018). Recent Advances in Metal Nanoparticles in Cancer Therapy. J. Drug Target..

[84] Li H., Zeng Y., Zhang H., Gu Z., Gong Q., Luo K. (2021). Functional Gadolinium-Based Nanoscale Systems for Cancer Theranostics. J. Control. Release.

[85] Li L., Tong R., Li M., Kohane D.S. (2016). Self-Assembled Gemcitabine–Gadolinium Nanoparticles for Magnetic Resonance Imaging and Cancer Therapy. Acta Biomater..

[86] Kotb S., Detappe A., Lux F., Appaix F., Barbier E.L., Tran V.-L., Plissonneau M., Gehan H., Lefranc F., Rodriguez-Lafrasse C. (2016). Gadolinium-Based Nanoparticles and Radiation Therapy for Multiple Brain Melanoma Metastases: Proof of Concept before Phase I Trial. Theranostics.

[87] Verry C., Dufort S., Lemasson B., Grand S., Pietras J., Troprès I., Crémillieux Y., Lux F., Mériaux S., Larrat B. (2020). Targeting Brain Metastases with Ultrasmall Theranostic Nanoparticles, a First-in-Human Trial from an MRI Perspective. Sci. Adv..

[88] Lammers T., Koczera P., Fokong S., Gremse F., Ehling J., Vogt M., Pich A., Storm G., van Zandvoort M., Kiessling F. (2015). Theranostic USPIO-Loaded Microbubbles for Mediating and Monitoring Blood-Brain Barrier Permeation. Adv. Funct. Mater..

[89] Deb S., Ghosh K., Shetty S. (2015). Nanoimaging in Cardiovascular Diseases: Current State of the Art. Indian J. Med. Res..

[90] Winter P.M., Neubauer A.M., Caruthers S.D., Harris T.D., Robertson J.D., Williams T.A., Schmieder A.H., Hu G., Allen J.S., Lacy E.K. (2006). Endothelial α_ν_ β_3_ Integrin–Targeted Fumagillin Nanoparticles Inhibit Angiogenesis in Atherosclerosis. ATVB.

[91] Kharaziha M., Memic A., Akbari M., Brafman D.A., Nikkhah M. (2016). Nano-Enabled Approaches for Stem Cell-Based Cardiac Tissue Engineering. Adv. Healthc. Mater..

[92] Yin R.-X., Yang D.-Z., Wu J.-Z. (2014). Nanoparticle Drug- and Gene-Eluting Stents for the Prevention and Treatment of Coronary Restenosis. Theranostics.

[93] Tsukie N., Nakano K., Matoba T., Masuda S., Iwata E., Miyagawa M., Zhao G., Meng W., Kishimoto J., Sunagawa K. (2013). Pitavastatin-Incorporated Nanoparticle-Eluting Stents Attenuate In-Stent Stenosis without Delayed Endothelial Healing Effects in a Porcine Coronary Artery Model. JAT.

[94] Leary S.P., Liu C.Y., Apuzzo M.L.J. (2006). Toward the Emergence of Nanoneurosurgery: Part III—Nanomedicine: Targeted Nanotherapy, Nanosurgery, and Progress Toward the Realization of Nanoneurosurgery. Neurosurgery.

[95] Shende P., Sardesai M., Gaud R.S. (2018). Micro to Nanoneedles: A Trend of Modernized Transepidermal Drug Delivery System. Artif. Cells Nanomed. Biotechnol..

[96] Zhu X., Kwok S.Y., Yuen M.F., Yan L., Chen W., Yang Y., Wang Z., Yu K.N., Zhu G., Zhang W. (2015). Dense Diamond Nanoneedle Arrays for Enhanced Intracellular Delivery of Drug Molecules to Cell Lines. J. Mater. Sci..

[97] Yamahata C., Collard D., Legrand B., Takekawa T., Kumemura M., Hashiguchi G., Fujita H. (2008). Silicon Nanotweezers With Subnanometer Resolution for the Micromanipulation of Biomolecules. J. Microelectromech. Syst..

[98] Chang W.C., Hawkes E.A., Kliot M., Sretavan D.W. (2007). In Vivo Use of a Nanoknife for Axon Microsurgery. Neurosurgery.

[99] Jourabchi N., Beroukhim K., Tafti B.A., Kee S.T., Lee E.W. (2014). Irreversible Electroporation (NanoKnife) in Cancer Treatment. Gastrointest. Interv..

[100] Singh S., Singh A. (2013). Current Status of Nanomedicine and Nanosurgery. Anesth. Essays Res..

[101] Zakrzewski W., Dobrzynski M., Dobrzynski W., Zawadzka-Knefel A., Janecki M., Kurek K., Lubojanski A., Szymonowicz M., Rybak Z., Wiglusz R.J. (2021). Nanomaterials Application in Orthodontics. Nanomaterials.

[102] Sun H., Lv L., Bai Y., Yang H., Zhou H., Li C., Yang L. (2018). Nanotechnology-Enabled Materials for Hemostatic and Anti-Infection Treatments in Orthopedic Surgery. IJN.

[103] Chen X., Schluesener H.J. (2008). Nanosilver: A Nanoproduct in Medical Application. Toxicol. Lett..

[104] Kanasty R., Dorkin J.R., Vegas A., Anderson D. (2013). Delivery Materials for SiRNA Therapeutics. Nat. Mater..

[105] Tabernero J., Shapiro G.I., LoRusso P.M., Cervantes A., Schwartz G.K., Weiss G.J., Paz-Ares L., Cho D.C., Infante J.R., Alsina M. (2013). First-in-Humans Trial of an RNA Interference Therapeutic Targeting VEGF and KSP in Cancer Patients with Liver Involvement. Cancer Discov..

[106] Jung J., Solanki A., Memoli K.A., Kamei K., Kim H., Drahl M.A., Williams L.J., Tseng H.-R., Lee K. (2010). Selective Inhibition of Human Brain Tumor Cells through Multifunctional Quantum-Dot-Based SiRNA Delivery. Angew. Chem. Int. Ed..

[107] Boca S., Gulei D., Zimta A.-A., Onaciu A., Magdo L., Tigu A.B., Ionescu C., Irimie A., Buiga R., Berindan-Neagoe I. (2020). Nanoscale Delivery Systems for MicroRNAs in Cancer Therapy. Cell. Mol. Life Sci..

[108] Wen M.M. (2016). Getting MiRNA Therapeutics into the Target Cells for Neurodegenerative Diseases: A Mini-Review. Front. Mol. Neurosci..

[109] Joga S., Koyyala V.P.B. (2021). Nanotechnology in Oncology. Indian J. Med. Paediatr. Oncol..

[110] Meng H., Liong M., Xia T., Li Z., Ji Z., Zink J.I., Nel A.E. (2010). Engineered Design of Mesoporous Silica Nanoparticles to Deliver Doxorubicin and P-Glycoprotein SiRNA to Overcome Drug Resistance in a Cancer Cell Line. ACS Nano.

[111] Meng H., Mai W.X., Zhang H., Xue M., Xia T., Lin S., Wang X., Zhao Y., Ji Z., Zink J.I. (2013). Codelivery of an Optimal Drug/SiRNA Combination Using Mesoporous Silica Nanoparticles to Overcome Drug Resistance in Breast Cancer in Vitro and in Vivo. ACS Nano.

[112] Brede C., Labhasetwar V. (2013). Applications of Nanoparticles in the Detection and Treatment of Kidney Diseases. Adv. Chronic Kidney Dis..

[113] Zhu X.-Y., Zou X., Mukherjee R., Yu Z., Ferguson C.M., Zhou W., McCollough C.H., Lerman L.O. (2018). Targeted Imaging of Renal Fibrosis Using Antibody-Conjugated Gold Nanoparticles in Renal Artery Stenosis. Investig. Radiol..

[114] Wang J., Masehi-Lano J.J., Chung E.J. (2017). Peptide and Antibody Ligands for Renal Targeting: Nanomedicine Strategies for Kidney Disease. Biomater. Sci..

[115] Williams R.M., Shah J., Tian H.S., Chen X., Geissmann F., Jaimes E.A., Heller D.A. (2018). Selective Nanoparticle Targeting of the Renal Tubules. Hypertension.

[116] Gong L., Wang Y., Liu J. (2017). Bioapplications of Renal-Clearable Luminescent Metal Nanoparticles. Biomater. Sci..

[117] Ordikhani F., Kasinath V., Uehara M., Akbarzadeh A., A Yilmam O., Dai L., Aksu H., Jung S., Jiang L., Li X. (2020). Selective Trafficking of Light Chain-Conjugated Nanoparticles to the Kidney and Renal Cell Carcinoma. Nano Today.

[118] Xue Y., Yu G., Shan Z., Li Z. (2018). Phyto-Mediated Synthesized Multifunctional Zn/CuO NPs Hybrid Nanoparticles for Enhanced Activity for Kidney Cancer Therapy: A Complete Physical and Biological Analysis. J. Photochem. Photobiol. B Biol..

[119] Minardi S., Shah S., Luo X. (2018). Biomimetic Nanoparticles for Transplantation Tolerance. Curr. Opin. Organ Transplant..

[120] Vemuri C., Upadhya G.A., Arif B., Jia J., Lin Y., Gaut J.P., Fazal J., Pan H., Wickline S.A., Chapman W.C. (2018). Antithrombin Perfluorocarbon Nanoparticles Improve Renal Allograft Function in a Murine Deceased Criteria Donor Model. Transplant. Direct..

[121] Uehara M., Bahmani B., Jiang L., Jung S., Banouni N., Kasinath V., Solhjou Z., Zhao J., Ordikhani F., Bae M. (2019). Nanodelivery of Mycophenolate Mofetil to the Organ Improves Transplant Vasculopathy. ACS Nano.

[122] Zhang Y., Pan J., Li H., Yu D., Wu T., Wang L., Wang Y., Zhou L., Zheng S. (2019). Albumin Based Nanomedicine for Enhancing Tacrolimus Safety and Lymphatic Targeting Efficiency. J. Biomed. Nanotechnol..

[123] Stead S.O., Kireta S., McInnes S.J.P., Kette F.D., Sivanathan K.N., Kim J., Cueto-Diaz E.J., Cunin F., Durand J.-O., Drogemuller C.J. (2018). Murine and Non-Human Primate Dendritic Cell Targeting Nanoparticles for in Vivo Generation of Regulatory T-Cells. ACS Nano.

[124] Brasile L., Henry N., Stubenitsky B. (2017). Underlying Mechanisms of Protection Involved in Immunocloak. Transplantation.

[125] Xie D., Xu Y., Jing W., Juxiang Z., Hailun L., Yu H., Zheng D.-H., Lin Y.-T. (2017). Berberine Nanoparticles Protects Tubular Epithelial Cells from Renal Ischemia-Reperfusion Injury. Oncotarget.

[126] Stephen Inbaraj B., Chen B.-H. (2020). An Overview on Recent in Vivo Biological Application of Cerium Oxide Nanoparticles. Asian J. Pharm. Sci..

[127] Chae E.Y., Song E.J., Sohn J.Y., Kim S.-T., Woo C.W., Gong G., Kang H.J., Lee J.S. (2010). Allogeneic Renal Graft Rejection in a Rat Model: In Vivo MR Imaging of the Homing Trait of Macrophages. Radiology.

[128] Loynachan C.N., Soleimany A.P., Dudani J.S., Lin Y., Najer A., Bekdemir A., Chen Q., Bhatia S.N., Stevens M.M. (2019). Renal Clearable Catalytic Gold Nanoclusters for in Vivo Disease Monitoring. Nat. Nanotechnol..

[129] Midgley A.C., Wei Y., Zhu D., Gao F., Yan H., Khalique A., Luo W., Jiang H., Liu X., Guo J. (2020). Multifunctional Natural Polymer Nanoparticles as Antifibrotic Gene Carriers for CKD Therapy. JASN.

[130] Kamaly N., He J.C., Ausiello D.A., Farokhzad O.C. (2016). Nanomedicines for Renal Disease: Current Status and Future Applications. Nat. Rev. Nephrol..

[131] Akhtar A.M., Schneider J.E., Chapman S.J., Jefferson A., Digby J.E., Mankia K., Chen Y., McAteer M.A., Wood K.J., Choudhury R.P. (2010). In Vivo Quantification of Vcam-1 Expression in Renal Ischemia Reperfusion Injury Using Non-Invasive Magnetic Resonance Molecular Imaging. PLoS ONE.

[132] Liu G.W., Pippin J.W., Eng D.G., Lv S., Shankland S.J., Pun S.H. (2020). Nanoparticles Exhibit Greater Accumulation in Kidney Glomeruli during Experimental Glomerular Kidney Disease. Physiol. Rep..

[133] Soriano M.L., Rodríguez-Benot A., Valcárcel M. (2018). Bases nanotecnológicas de una «nueva» Nefrología. Nefrología.

[134] Martuszewski A., Paluszkiewicz P., Król M., Banasik M., Kepinska M. (2021). Donor-Derived Cell-Free DNA in Kidney Transplantation as a Potential Rejection Biomarker: A Systematic Literature Review. JCM.

[135] Stanimirova I., Banasik M., Ząbek A., Dawiskiba T., Kościelska-Kasprzak K., Wojtowicz W., Krajewska M., Janczak D., Młynarz P. (2020). Serum Metabolomics Approach to Monitor the Changes in Metabolite Profiles Following Renal Transplantation. Sci. Rep..

[136] Terasaki P.I. (2003). Humoral Theory of Transplantation: Humoral Theory of Transplantation. Am. J. Transplant..

[137] Klinger M., Banasik M. (2015). Immunological Characteristics of the Elderly Allograft Recipient. Transplant. Rev..

[138] Banasik M., Boratyńska M., Kościelska-Kasprzak K., Kamińska D., Bartoszek D., Żabińska M., Myszka M., Zmonarski S., Protasiewicz M., Nowakowska B. (2014). The Influence of Non-HLA Antibodies Directed against Angiotensin II Type 1 Receptor (AT1R) on Early Renal Transplant Outcomes. Transpl. Int..

[139] Tietjen G.T., Hosgood S.A., DiRito J., Cui J., Deep D., Song E., Kraehling J.R., Piotrowski-Daspit A.S., Kirkiles-Smith N.C., Al-Lamki R. (2017). Nanoparticle Targeting to the Endothelium during Normothermic Machine Perfusion of Human Kidneys. Sci. Transl. Med..

[140] Hosgood S.A., Hoff M., Nicholson M.L. (2021). Treatment of Transplant Kidneys during Machine Perfusion. Transpl. Int..

[141] DiRito J.R., Hosgood S.A., Tietjen G.T., Nicholson M.L. (2018). The Future of Marginal Kidney Repair in the Context of Normothermic Machine Perfusion. Am. J. Transpl..

[142] DiRito J.R., Hosgood S.A., Reschke M., Albert C., Bracaglia L.G., Ferdinand J.R., Stewart B.J., Edwards C.M., Vaish A.G., Thiru S. (2021). Lysis of Cold-storage-induced Microvascular Obstructions for Ex Vivo Revitalization of Marginal Human Kidneys. Am. J. Transpl..

[143] Feng S., Zhou L., Lin D., Zhao J., Guan Q., Zheng B., Wang K., Li H., Chen R., Zeng H. (2019). Assessment of Treatment Efficacy Using Surface-Enhanced Raman Spectroscopy Analysis of Urine in Rats with Kidney Transplantation or Kidney Disease. Clin. Exp. Nephrol..

[144] Chi J., Ma Y., Weng F.L., Thiessen-Philbrook H., Parikh C.R., Du H. (2017). Surface-Enhanced Raman Scattering Analysis of Urine from Deceased Donors as a Prognostic Tool for Kidney Transplant Outcome. J. Biophotonics.

[145] Chen Y., Han X., Sun Y., He X., Xue D. (2020). A Circulating Exosomal MicroRNA Panel as a Novel Biomarker for Monitoring Post-transplant Renal Graft Function. J. Cell. Mol. Med..

[146] Kasiske B.L., Zeier M.G., Chapman J.R., Craig J.C., Ekberg H., Garvey C.A., Green M.D., Jha V., Josephson M.A., Kiberd B.A. (2010). KDIGO Clinical Practice Guideline for the Care of Kidney Transplant Recipients: A Summary. Kidney Int..

[147] (2009). Special Issue: KDIGO Clinical Practice Guideline for the Care of Kidney Transplant Recipients. Am. J. Transplant..

[148] Josephson M.A. (2011). Monitoring and Managing Graft Health in the Kidney Transplant Recipient. CJASN.

[149] Benoît G. (2011). Surgical view of a series of 3000 kidney transplantations. Bull. Acad. Natl. Med..

[150] Carvalho J.A., Nunes P., Antunes H., Parada B., Tavares da Silva E., Rodrigues L., Roseiro A., Bastos C., Macário F., Figueiredo A. (2019). Surgical Complications in Kidney Transplantation: An Overview of a Portuguese Reference Center. Transpl. Proc..

[151] Hakim D.N., Nader M.A., Sood A., Kandilis A., Hakim N.S. (2016). Rescue of Transplanted Kidney Thanks to an Implantable Doppler Probe: Is This the Future?. Exp. Clin. Transplant..

[152] Rodgers S.K., Sereni C.P., Horrow M.M. (2014). Ultrasonographic Evaluation of the Renal Transplant. Radiol. Clin. N. Am..

[153] Malakasioti G., Marks S.D., Watson T., Williams F., Taylor-Allkins M., Mamode N., Morgan J., Hayes W.N. (2018). Continuous Monitoring of Kidney Transplant Perfusion with Near-Infrared Spectroscopy. Nephrol. Dial. Transplant..

[154] Thongprayoon C., Hansrivijit P., Leeaphorn N., Acharya P., Torres-Ortiz A., Kaewput W., Kovvuru K., Kanduri S., Bathini T., Cheungpasitporn W. (2020). Recent Advances and Clinical Outcomes of Kidney Transplantation. JCM.

[155] Chadban S.J., Wu H., Hughes J. (2010). Macrophages and Kidney Transplantation. Semin. Nephrol..

[156] Islam T., Wolf G. (2009). The Pharmacokinetics of the Lymphotropic Nanoparticle MRI Contrast Agent Ferumoxtran-10. CBM.

[157] Aghighi M., Pisani L., Theruvath A.J., Muehe A.M., Donig J., Khan R., Holdsworth S.J., Kambham N., Concepcion W., Grimm P.C. (2018). Ferumoxytol Is Not Retained in Kidney Allografts in Patients Undergoing Acute Rejection. Mol. Imaging Biol..

[158] Yang D., Ye Q., Williams M., Sun Y., Hu T.C.-C., Williams D.S., Moura J.M.F., Ho C. (2001). USPIO-Enhanced Dynamic MRI: Evaluation of Normal and Transplanted Rat Kidneys. Magn. Reson. Med..

[159] Hauger O., Grenier N., Deminère C., Lasseur C., Delmas Y., Merville P., Combe C. (2007). USPIO-Enhanced MR Imaging of Macrophage Infiltration in Native and Transplanted Kidneys: Initial Results in Humans. Eur. Radiol..

[160] Halloran P.F., Urmson J., Ramassar V., Melk A., Zhu L.-F., Halloran B.P., Bleackley R.C. (2004). Lesions of T-Cell-Mediated Kidney Allograft Rejection in Mice Do Not Require Perforin or Granzymes A and B. Am. J. Transplant..

[161] Ramanathan A. (2019). Toxicity of Nanoparticles_ Challenges and Opportunities. Appl. Microsc..

[162] Bozorgi A., Khazaei M., Soleimani M., Jamalpoor Z. (2021). Application of Nanoparticles in Bone Tissue Engineering; a Review on the Molecular Mechanisms Driving Osteogenesis. Biomater. Sci..

